# Alpha hemolysin enhances the immune response by modulating dendritic cell differentiation via ADAM10-Notch signaling

**DOI:** 10.1038/s41392-025-02432-3

**Published:** 2025-10-08

**Authors:** Ke Wang, Jingwen Liao, Yue Yuan, Zhifu Chen, Qiang Gou, Haiming Jing, Mengmeng Liang, Yuanda Tang, Pengju Yan, Xiaoqian Yu, Zhuo Zhao, Tianjun Sun, Zhenping Xia, Ting Yu, Yaling Liao, Hao Zeng, Xiaoli Zhang, Quanming Zou, Jinyong Zhang

**Affiliations:** 1 National Engineering Research Center of Immunological Products, Department of Microbiology and Biochemical Pharmacy, College of Pharmacy, Army Medical University, Chongqing, China; 2 College of Bioengineering, Chongqing University, Chongqing, China; 3 Institute of Cancer, Xinqiao Hospital, Army Medical University, Chongqing, China; 4 Chongqing Key Laboratory of Immunotherapy, Chongqing, China; 5 Department of Orthopedics, Southwest Hospital, Army Medical University, Chongqing, China; 6 Tianjin Institute of Environmental and Operational Medicine, Tianjin, China; 7 Department of Clinical Laboratory, The 89 Hospital of The People’s Liberation Army, Weifang, China; 8 Department of Clinical Hematology, College of Pharmacy, Army Medical University, Chongqing, China

**Keywords:** Vaccines, Infectious diseases

## Abstract

Alpha hemolysin, a pore-forming toxin from *Staphylococcus aureus*, is a critical virulence factor for bacteria. Previous studies have demonstrated that the Hla mutant H35A (Hla_H35A_) serves as a potent carrier protein for subunit vaccines, yet its immunomodulatory mechanisms remain incompletely understood. Here, we demonstrate that the Hla_H35A_ fusion enhances vaccine efficacy by targeting A Disintegrin and Metalloproteinase 10 (ADAM10) on dendritic cells (DCs), thereby activating the ADAM10-Notch signaling axis. Using the candidate antigen PA0833 from *Pseudomonas aeruginosa* as a model, we show that the Hla_H35A_-PA0833 fusion protein (HPF) significantly augments antigen uptake, DC maturation, and Notch-dependent transcriptional programs, particularly in conventional DCs (cDCs). The Hla_H35A_ fusion drives the differentiation of Notch2-dependent cDC2s, which is marked by ESAM expression and IL-23 secretion. This process promotes Th17 and T follicular helper (Tfh) cell responses in draining lymph nodes, leading to elevated antigen-specific IgG1 titers and robust protection against acute *Pseudomonas aeruginosa* lung infection. Notably, ADAM10 or Notch inhibition abrogates these effects. Similarly, human monocyte-derived DCs exhibit enhanced maturation and Notch activation via the Hla_H35A_-ADAM10 interaction. Our findings reveal that Hla_H35A_ is a novel carrier protein that shapes adaptive immunity by modulating cDC2 differentiation via ADAM10-Notch2 signaling, suggesting a promising strategy for Th17/Tfh-oriented vaccine design.

## Introduction

*Staphylococcus aureus* alpha hemolysin (Hla/α-toxin) is a pore-forming cytotoxin that plays a key role in pathogenesis.^1^ During infection, Hla binds to susceptible host cells and forms transmembrane pores, initiating programmed cell death.^2^ A Disintegrin and Metalloproteinase 10 (ADAM10) has been identified as the critical host receptor mediating this cytotoxic activity, and its interaction is essential for bacterial virulence.^3^ ADAM10, an I-type transmembrane protein, is extensively expressed in human antigen-presenting cells, making Hla a promising target for vaccine development. Pre-clinical studies have shown that both active and passive immunization with Hla offer partial protection against *Staphylococcus aureus* infection.^4,5^ While wild-type Hla exhibits potent hemolytic activity, engineered Hla mutants (e.g., H35A) retain immunogenicity and ADAM10-binding capacity despite the loss of pore-forming function.^6–8^ In addition, our previous studies demonstrated that the Hla mutant H35A (Hla_H35A_) functions as an effective carrier protein, significantly enhancing antigen-specific antibody responses and protective immunity.^9^ Its synergistic effect with aluminum adjuvants further enhances the immune response, suggesting a mechanism distinct from traditional aluminum adjuvants.^9^ One potential mechanism is that the interaction between Hla_H35A_ and ADAM10 facilitates antigen acquisition and presentation by macrophages.^9^ However, the downstream signaling pathways and broader immunomodulatory effects of this interaction remain incompletely characterized.

The ADAM family comprises transmembrane endopeptidases that regulate diverse biological processes via proteolytic cleavage of multiple substrates.^10^ Among them, ADAM10 and ADAM17 have a close phylogenetic relationship and are widely expressed by most immune cells. In lymphoid cells, ADAM10 and ADAM17 play crucial roles as sheddases in regulating T cell activation and proliferation, B cell development, and maintaining the structural integrity of lymphoid organs.^11^ In myeloid cells, ADAM10 and ADAM17 are essential shedding enzymes that regulate transendothelial migration, cytokine production, and intracellular signaling cascades.^11^ Notably, ADAM10-mediated Notch receptor cleavage critically governs immune cell differentiation and fate determination, particularly in T cells, marginal zone B cells, and dendritic cells (DCs).^11^ Typically, mature Notch receptors on the cell membrane exist as heterodimers, with their heterodimerization domain undergoing S1 cleavage in the Golgi apparatus, forming a Ca^2+^-stabilized heterodimer.^12^ Ligand binding to the extracellular domain of Notch triggers ligand endocytosis, which induces a conformational change in the receptor, exposing the S2 cleavage site.^12^ Upon sequential S2 cleavage by ADAM10 and S3 cleavage by γ-secretase, Notch releases the Notch intracellular domain (NICD), which then translocates to the nucleus and forms a transcriptional activation complex with RBPJ and MAML1 to regulate Notch-responsive genes.^12^ Nevertheless, how exogenous ADAM10 ligands, including antigens, modulate immune cell function, especially in antigen-presenting cells, requires further investigation.

ADAM10 is constitutively and abundantly expressed on antigen-presenting cells (APCs), particularly DCs.^11^ Given these findings, we hypothesize that the Hla_H35A_-ADAM10 interaction plays a pivotal role in DC activation and differentiation, which are crucial for vaccine efficacy. Although derived from hematopoietic precursors, DCs display remarkable phenotypic and functional heterogeneity. Unlike monocyte-derived DCs (MoDCs) and plasmacytoid DCs (pDCs), conventional DCs (cDCs), the predominant subset, are derived from fms-like tyrosine kinase 3 ligand (Flt3L)-dependent DC precursors (pre-DCs) and differentiate into cDC1s and cDC2s in peripheral tissues and lymphoid organs.^13^ cDC1s, characterized by Xcr1 expression, specialize in cross-presenting antigens to CD8^+^ T cells for intracellular pathogen defense.^14^ In contrast, cDC2s express CD11b and SIRPα, engaging CD4^+^ T cells to elicit T helper (Th) and T follicular helper (Tfh) responses. cDC2s are highly heterogeneous, classified into subsets on the basis of various criteria and perform nonredundant functions, particularly in Th2 and Th17 immunity.^14^ Given their central role in T-cell priming, DCs critically affect vaccine outcomes, as different formulations elicit divergent T-cell responses.^15^ For example, saponins modulate Th1/Th2 polarization through DCs, although their receptors remain unidentified.^16^ While traditional vaccine carriers focus more on enhancing immune effects through pattern recognition receptors (PRRs), understanding how vaccine components regulate DC differentiation and function is essential for optimizing immunization strategies and directing immune responses.

In this study, we employed PA0833, an OmpA-like protein identified as a novel protective antigen from *Pseudomonas aeruginosa* (*P. aeruginosa*), as the target antigen. By fusing PA0833 with the Hla_H35A_ carrier protein to generate the Hla_H35A_-PA0833 fusion antigen (HPF), we established a model system to investigate the impact of Hla_H35A_ fusion on DCs through in vitro cellular experiments and in vivo mouse models. Our findings revealed that ADAM10 is a key target of Hla_H35A_ and plays a crucial role in DC maturation. Specifically, we found that Notch signaling activation mediated by Hla_H35A_-ADAM10 significantly impacts the maturation and differentiation of cDCs but not MoDCs. Moreover, in vivo experiments demonstrated that Hla_H35A_ fusion significantly enhances Th17 and Tfh responses by activating the ADAM10-Notch signaling pathway in cDCs. Additionally, we observed that the activation of ADAM10-Notch signaling in cDCs leads to an increased antibody response against PA0833 and provides protection against lung injury induced by acute infection with the *P. aeruginosa* strain PAO1. Our findings emphasize the importance of Hla_H35A_ as a novel carrier protein and intramolecular adjuvant in subunit vaccine design.

## Results

### ADAM10 mediates Hla_H35A_-enhanced antigen immunogenicity and protective efficacy

**Fig. 1:**
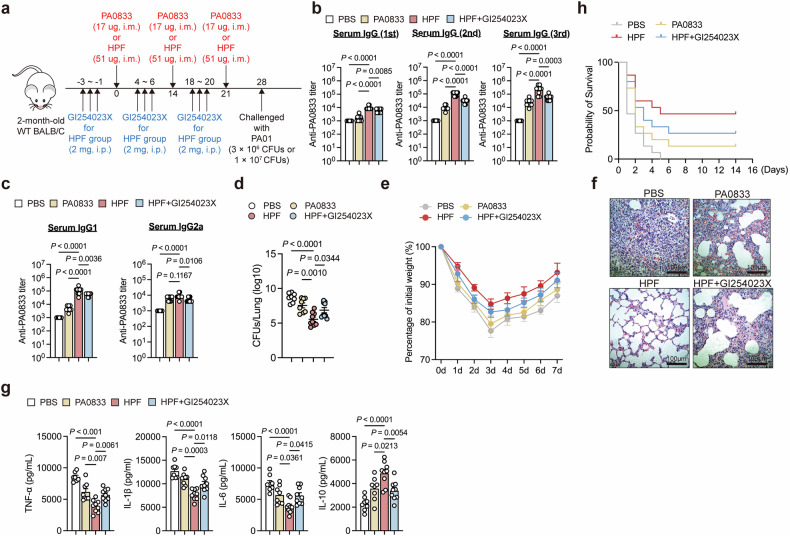
Hla_H35A_ improves the antibody titer and protection efficacy of antigens carried via ADAM10. **a** Experimental design for the mouse immunization model. **b** Serum levels of PA0833-specific IgG in mice immunized with PBS, PA0833, HPF or HPF with GI254023X after three immunizations (*n* = 6–10 per group). The data were pooled from two independent experiments. **c** Serum levels of PA0833-specific IgG1 or IgG2a in mice immunized with PBS, PA0833, HPF or HPF with GI254023X treatment (*n* = 6–10 per group). The data were pooled from two independent experiments. **d** Bacterial loads in the lungs were determined 48 h after challenge with a sublethal dose (3 × 10^6^ CFU) of PAO1 in mice immunized with PBS, PA0833, HPF or HPF with GI254023X treatment (*n* = 6–10 per group). The data were pooled from two independent experiments. **e** Body weight was measured 7 d after challenge with a sublethal dose (3 × 10^6^ CFU) of PAO1 in mice immunized with PBS, PA0833, HPF or HPF with GI254023X treatment (*n* = 7 per group). **f** Representative H&E staining of lung sections obtained 48 h after challenge with a sublethal dose (3 × 10^6^ CFU) of PAO1 from mice immunized with PBS, PA0833, HPF or HPF with GI254023X treatment (*n* = 3 per group). **g** TNF-α, IL-1β, IL-6, and IL-10 levels in the lungs were determined 48 h after challenge with a sublethal dose (3 × 10^6^ CFU) of PAO1 in mice immunized with PBS, PA0833, HPF or HPF with GI254023X treatment (*n* = 6–10 per group). The data were pooled from two independent experiments. **h** Kaplan–Meier survival curves of mice challenged with a lethal dose (1 × 10⁷ CFU) of PAO1, measured 14 days post-infection, following immunization with PBS, PA0833, HPF, or HPF with GI254023X treatment (*n* = 15 per group). Each data point indicates a biological replicate in (**b**–**d**) and (**g**). The data are presented as the means ± s.e.m.s. Statistical significance was determined via one-way ANOVA followed by Tukey’s multiple comparisons test in (**b**–**d**) and (**g**). H&E staining hemoglobin and eosin staining, CFU colony forming unit

Research has shown that the interaction between Hla_H35A_ and ADAM10 on immune cells is crucial for enhancing immune protection against acute lung infection caused by *P. aeruginosa*.^9^ To further investigate the significance of ADAM10 in the protective efficacy of Hla_H35A_ fusion proteins, mice were immunized three times with HPF. One week after the third immunization, the mice were challenged with 3 × 10^6^ or 1 × 10^7^ CFU of *P. aeruginosa* strain PAO1 (Fig. 1a). Consistent with our previous studies, Hla_H35A_ fusion significantly increased the titer of PA0833-specific IgG compared with that of PA0833 alone, whereas this increase was significantly attenuated by the ADAM10-specific inhibitor GI254023X (Fig. 1b). Intriguingly, Hla_H35A_ fusion selectively increased IgG1 titers but not IgG2a titers, whereas systemic ADAM10 inhibition reduced both subclasses (Fig. 1c), indicating that ADAM10 may perform distinct functions across different immune cell types.

To further investigate the role of ADAM10 in vaccine-induced immune protection, we infected immunized mice with a sublethal dose of PAO1 and evaluated the bacterial burden in the lungs. Compared with PA0833 alone, HPF immunization markedly reduced the number of lung PAO1 CFUs, and this effect was abrogated by ADAM10 inhibition (Fig. 1d). Concurrently, we monitored body weight changes for one week post-challenge. All groups exhibited rapid weight loss (days 0–3) followed by gradual recovery, with Hla_H35A_ fusion-immunized mice showing minimal decline (Fig. 1e). Histological analysis demonstrated attenuated lung inflammation in Hla_H35A_ fusion-treated mice compared with PA0833 controls, whereas ADAM10 blockade compromised this protection (Fig. 1f). Moreover, cytokine profiling revealed that Hla_H35A_ fusion significantly suppressed the expression of proinflammatory mediators (TNF-α, IL-1β, and IL-6) while increasing the expression of the anti-inflammatory cytokine IL-10, and treatment with an ADAM10 inhibitor reversed these changes (Fig. 1g). Finally, in lethal challenge studies (1 × 10^7^ CFU), HPF immunization resulted in 46% survival, whereas ADAM10 inhibition reduced protection to 26%, and PA0833 alone resulted in only 13% survival (Fig. 1h). These findings highlight the critical role of ADAM10 in mediating the immune-enhancing properties of Hla_H35A_.

### Transcriptional features of Hla_H35A_-stimulated BMDCs

**Fig. 2:**
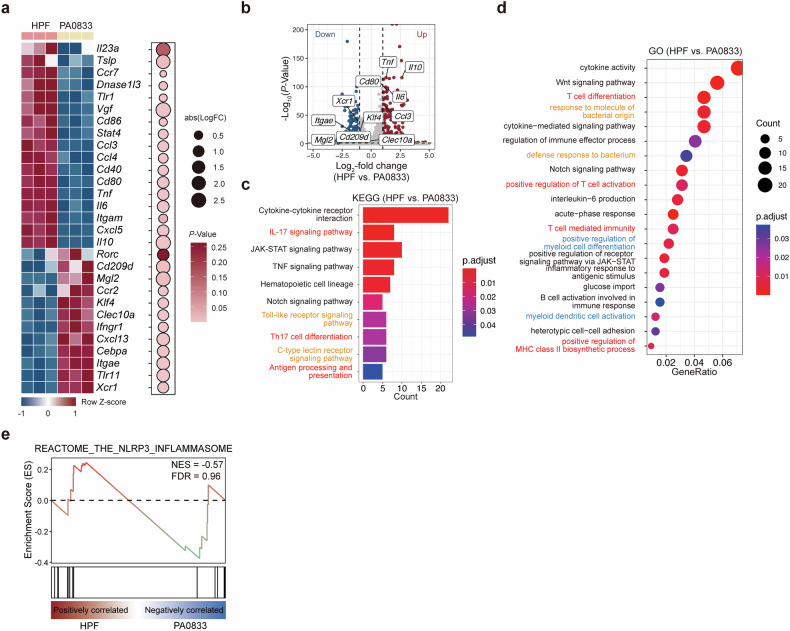
Bulk RNA-seq analysis of Hla_H35A_ treated Fl-BMDCs compared with control Fl-BMDCs subjected to antigen treatment. **a** Heatmap of representative gene expression in Fl-BMDCs after 7.5 h of treatment with HPF or PA0833. The relative expression abundance (rowwise Z score of the log2(TPM + 1), where TPMs denote transcripts per million; color scale) of genes (rows) across conditions (columns) is shown. **b** DEGs (|log2-fold change| > 1 and *P* value < 0.05) in Fl-BMDCs treated with HPF vs. PA0833. Upregulated genes: 257; downregulated genes: 307. **c** KEGG analysis of upregulated genes in Fl-BMDCs after 7.5 h of treatment with HPF vs. PA0833. Red indicates T-cell response-related signals, and orange indicates PRR-related signals. **d** GO enrichment analysis of upregulated genes in Fl-BMDCs after 7.5 h of treatment with HPF or PA0833. Red indicates T-cell response-related signals, blue indicates dendritic cell development-related signals, and orange indicates PRR-related signals. **e** GSEA showing enrichment of the REACTOME_THE_NLRP3_INFLAMMASOME pathway in Fl-BMDCs treated with HPF vs. PA0833. DEG differentially expressed gene

DCs play a crucial role in the immune response by connecting innate and adaptive immunity through antigen presentation,^14^ and cDCs constitute the primary subset of DCs found in both lymphoid and nonlymphoid tissues. To characterize Hla_H35A_-specific transcriptional profiling in cDCs, we conducted bulk RNA-seq on Flt3L-derived BMDCs (Fl-BMDCs) stimulated with HPF or PA0833, with PBS as an antigen unstimulated control. Principal component analysis (PCA) revealed distinct transcriptional changes in Fl-BMDCs following different antigen treatments, with Hla_H35A_ fusion inducing more pronounced differentially expressed genes (DEGs) (Supplementary Fig. 1a–c). Following PA0833 and HPF stimulation, genes associated with costimulatory molecules (e.g., *Cd40, Cd80*, and *Cd86*) and inflammatory factors (e.g., *Tnf*, *Il6*, and *Il10*) were significantly upregulated compared with those in the unstimulated group (Supplementary Fig. 1d). Importantly, compared with PA0833 alone, the Hla_H35A_ fusion further increased the expression of these genes, indicating enhanced DC maturation and inflammatory activation (Fig. 2a, b).

Kyoto Encyclopedia of Genes and Genomes (KEGG) analysis of the differentially expressed genes revealed enrichment in PRR signaling, T-cell responses, and inflammatory pathways in both PA0833- and HPF-treated Fl-BMDCs (Supplementary Fig. 1e). Notably, compared with PA0833 alone, Hla_H35A_ fusion specifically enhanced PRR- and T-cell-related pathways, particularly Th17 responses (Fig. 2c). Gene Ontology (GO) analysis corroborated these findings, revealing that Hla_H35A_ fusion amplified pathway activation beyond PA0833 stimulation (Fig. 2d and Supplementary Fig. 1f). Furthermore, the Hla_H35A_ fusion exhibited superior efficacy in promoting BMDC maturation and differentiation. Although high-purity recombinant *S. aureus* α-toxin is known to activate NLRP3 inflammasome-mediated pyroptosis,^17^ gene set enrichment analysis (GSEA) confirmed that Hla_H35A_ fusion does not trigger this pathway (Fig. 2e). Collectively, these data indicate that Hla_H35A_ fusion predominantly modulates DC maturation, cytokine production, and subsequent T-cell activation without engaging in NLRP3 inflammasome signaling.

### ADAM10 mediates Hla_H35A_-dependent DC maturation

Given the pivotal role of ADAM10 in Hla_H35A_ fusion-induced immune protection, we hypothesized that this interaction is equally critical for dendritic cells (DCs). To test this hypothesis, we first assessed the in vivo impact of the Hla_H35A_ fusion protein on DCs. The number of DCs significantly increased in both the dLN and injection sites 1 day post-immunization, and Hla_H35A_ fusion significantly increased the number of DCs at these two sites. Notably, ADAM10 inhibition not only led to diminished functionality of the Hla_H35A_ fusion antigen 1 day post-immunization but also appeared to induce its accumulation at the injection site 3 days post-immunization (Supplementary Fig. 2a, b).

**Fig. 3:**
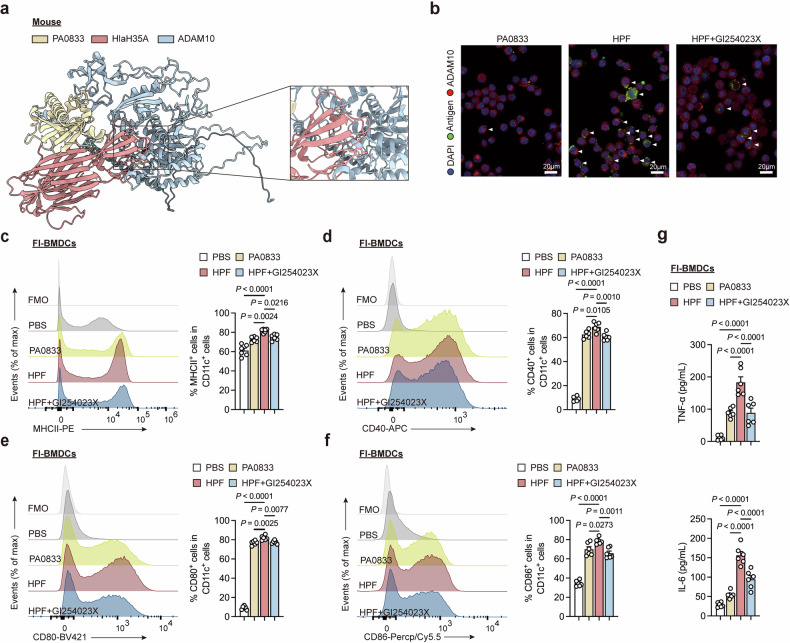
Hla_H35A_ promotes Fl-BMDC maturation via ADAM10. **a** Predicted accurate model of the interaction of HPFs with mouse ADAM10 (pLDDT = 72.7). The predicted local distance difference test score (pLDDT) was used to evaluate per-residue confidence, with values ≥ 70 indicating reliable backbone modeling. **b** Representative fluorescence images showing interactions of PA0833 or HPF with ADAM10 on the surface of Fl-BMDCs after 6 h of treatment with PA0833, HPF, or HPF with GI254023X (*n* = 3 per group). The antigens used were PA0833 or HPF. The arrowheads indicate cells that captured the antigens. **c** Representative flow cytometry histogram (left) and quantification (right) of MHCII^+^ cell frequencies gated on CD11c^+^ cells from Fl-BMDCs after 7.5 h of treatment with PBS, PA0833, HPF, or HPF with GI254023X (*n* = 6 per group). The data were pooled from two independent experiments. **d** Representative flow cytometry histogram (left) and quantification (right) of CD40^+^ cell frequencies gated on CD11c^+^ cells from Fl-BMDCs after 7.5 h of treatment with PBS, PA0833, HPF, or HPF with GI254023X (*n* = 6 per group). The data were pooled from two independent experiments. **e** Representative flow cytometry histogram (left) and quantification (right) of CD80^+^ cell frequencies gated on CD11c^+^ cells from Fl-BMDCs after 7.5 h of treatment with PBS, PA0833, HPF, or HPF with GI254023X (*n* = 6 per group). The data were pooled from two independent experiments. **f** Representative flow cytometry histogram (left) and quantification (right) of CD86^+^ cell frequencies gated on CD11c^+^ cells from Fl-BMDCs after 7.5 h of treatment with PBS, PA0833, HPF, or HPF with GI254023X (*n* = 6 per group). The data were pooled from two independent experiments. **g** Serum levels of TNF-α or IL-6 secreted from Fl-BMDCs after 7.5 h of treatment with PBS, PA0833, HPF, or HPF with GI254023X (*n* = 6 per group). The data were pooled from two independent experiments. Each data point indicates a biological replicate in (**c**–**g**). The data are presented as the means ± s.e.m.s. Statistical significance was tested via one-way ANOVA followed by Tukey’s multiple comparisons test in (**c**–**g**). Fl-BMDCs Flt3L-induced bone marrow dendritic cells, pLDDT predicted local-distance difference test

To further characterize this mechanism, we generated two DC subsets in vitro, one using Flt3L to generate conventional DCs (cDCs) and plasmacytoid DCs (pDCs) and the other combining GM-CSF and IL-4 to produce monocyte-derived DCs (MoDCs). Treatment with PA0833, HPF, or HPF following pre-treatment with the ADAM10 inhibitor GI254023X resulted in no cytotoxicity (Supplementary Fig. 3a, b). Structural predictions (AlphaFold-Multimer) and fluorescence confocal microscopy confirmed Hla_H35A_-specific ADAM10 binding (Fig. 3a, b). This interaction significantly increased antigen uptake by DCs, which is consistent with our previous observations in RAW264.7 cells.^9^ These results demonstrate the critical role of ADAM10 in Hla_H35A_-mediated antigen uptake by DCs.

To assess ADAM10-dependent Hla_H35A_ fusion-mediated DC maturation, we analyzed the surface expression of MHC-II, CD40, CD80, and CD86 via flow cytometry (Supplementary Fig. 4a, b). Consistent with the results of the transcriptional profile, Hla_H35A_ fusion significantly enhanced the maturation of both Fl-BMDCs and Mo-BMDCs in an ADAM10-dependent manner (Fig. 3c–f, Supplementary Fig. 4c–f). Additionally, Hla_H35A_ fusion significantly increased inflammatory cytokine secretion through ADAM10, including TNF-α and IL-6, in Fl-BMDCs and IL-6 in Mo-BMDCs (Fig. 3g and Supplementary Fig. 4g). Importantly, Mo-BMDCs produced higher levels of IL-6 than did Fl-BMDCs, which aligns with the established role of MoDCs in T-cell activation via inflammatory mediators.^18^ Collectively, these data demonstrate that the Hla_H35A_ fusion promotes DC maturation and cytokine production through ADAM10.

### Hla_H35A_ activates Notch signaling in DCs in an ADAM10-dependent manner

**Fig. 4:**
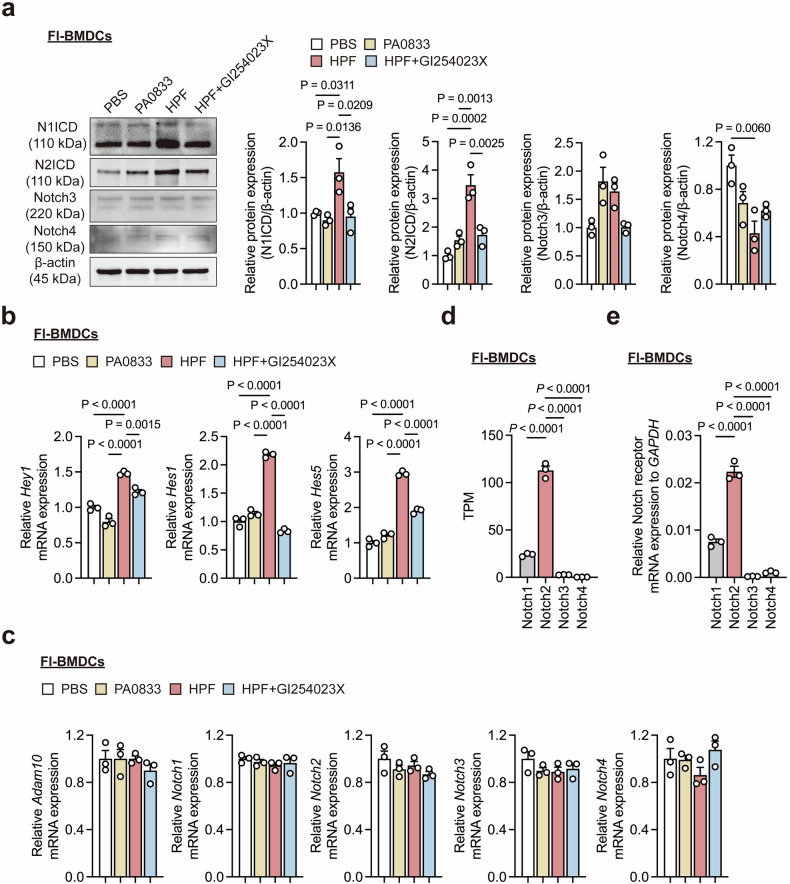
Hla_H35A_ activates Notch signaling via ADAM10 in Fl-BMDCs. **a** Representative western blot (left) and quantification (right) of N1ICD, N2ICD, Notch3, or Notch4 in Fl-BMDCs after 4.5 h of treatment with PBS, PA0833, HPF, or HPF with GI254023X (*n* = 3 per group). **b** Quantification of *Hey1*, *Hes1*, or *Hes5* mRNA expression by qPCR in Fl-BMDCs after 6 h of treatment with PBS, PA0833, HPF, or HPF with GI254023X (*n* = 3 per group). **c** Quantification of *Adam10*, *Notch1*, *Notch2*, *Notch3*, or *Notch4* mRNA expression by qPCR in Fl-BMDCs after 4.5 h of treatment with PBS, PA0833, HPF, or HPF with GI254023X (*n* = 3 per group). **d** TPMs of the *Notch1*, *Notch2*, *Notch3*, or *Notch4* genes in unstimulated Fl-BMDCs from bulk RNA-seq (*n* = 3 per group)**. e** Quantification of *Notch1*, *Notch2*, *Notch3*, or *Notch4* mRNA expression by qPCR in Fl-BMDCs under homeostatic conditions (*n* = 3 per group). Each data point indicates a biological replicate in (**a**) and (**d**). Each data point indicates a biological replicate derived from the average of three technical replicates in (**b**, **c** and **e**). The data are presented as the means ± s.e.m.s. Statistical significance was tested via one-way ANOVA followed by Tukey’s multiple comparisons test in (**a**–**e**)

In addition to serving as a receptor for Hla, ADAM10 plays crucial roles in immune cell development.^11^ Transcriptomic analysis revealed that compared with PA0833, the Hla_H35A_ fusion protein HPF significantly upregulated Notch signaling in BMDCs (Fig. 2c, d), suggesting that the Hla_H35A_-ADAM10 interaction may mediate Notch activation (Supplementary Fig. 5a). As expected, our results showed that Hla_H35A_ specifically enhanced ADAM10-dependent cleavage of Notch1/2 intracellular domains (N1ICD/N2ICD) in Fl-BMDCs compared with PA0833, without affecting Notch3/4 levels (Fig. 4a). Correspondingly, Hla_H35A_ fusion upregulated the expression of Notch1/2 target genes (*Hes1*, *Hes5*, and *Hey1*) in an ADAM10-dependent manner (Fig. 4b).

To investigate the broader effects of Hla_H35A_ fusion on different DC subsets, we examined the impact of HPF and PA0833 on Mo-BMDCs. Similar to the results observed in Fl-BMDCs, Hla_H35A_ fusion triggered ADAM10-dependent Notch1/2 signaling in Mo-BMDCs (Supplementary Fig. 5b, c). In contrast, stimulation of Fl-BMDCs with PA0833 and HPF did not lead to a significant increase in ADAM10 or Notch mRNA levels (Fig. 4c), indicating that Hla_H35A_ fusion activates ADAM10-dependent Notch1/2 signaling through receptor cleavage rather than transcriptional upregulation. Notably, the expression of Notch2 in Fl-BMDCs was higher than that of other Notch receptors (Fig. 4d, e). These results suggest that Hla_H35A_ mediates the cleavage of Notch receptors via ADAM10, leading to the activation of downstream Notch1/2 signaling in both MoDCs and cDCs.

### Hla_H35A_ mediates Notch2-dependent cDC2 differentiation via ADAM10

**Fig. 5:**
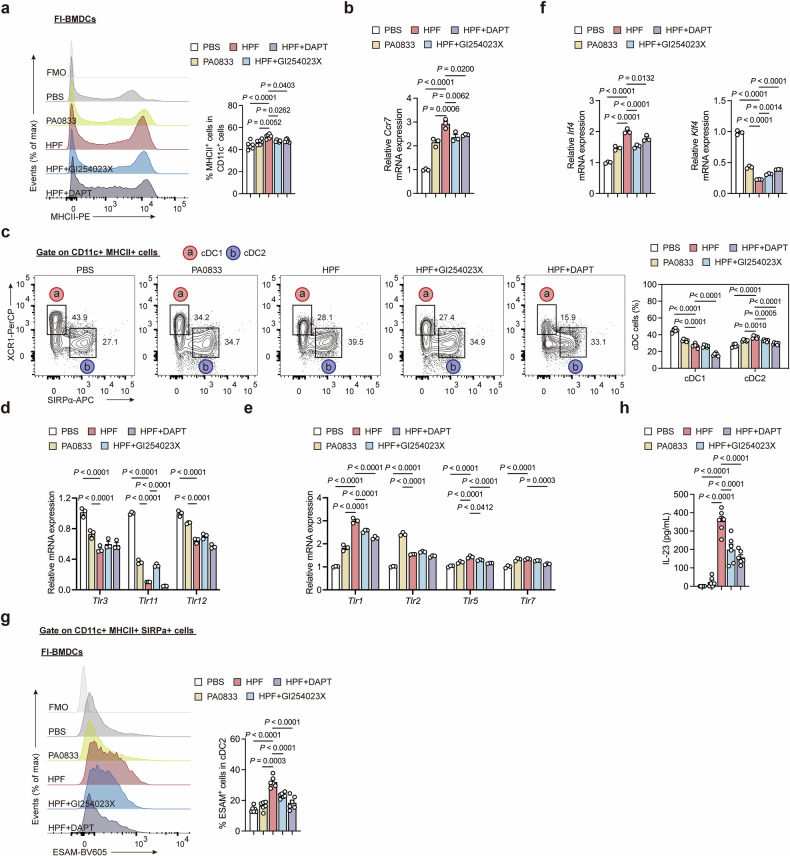
Hla_H35A_ induces Notch2-dependent cDC2s via ADAM10-Notch signaling. **a** Representative flow cytometry histogram (left) and quantification (right) of MHCII^+^ cell frequencies gated on CD11c^+^ cells from Fl-BMDCs after 7.5 h of treatment with PBS, PA0833, HPF, HPF with GI254023X or HPF with DAPT (*n* = 6 per group). The data were pooled from two independent experiments. **b** Quantification of *Ccr7* mRNA expression by qPCR in Fl-BMDCs after 7.5 h of treatment with PBS, PA0833, HPF, HPF with GI254023X or HPF with DAPT (*n* = 3). **c** Representative flow cytometry plots (left) and quantification (right) of cDC subset frequencies in Fl-BMDCs after 12 h of treatment with PBS, PA0833, HPF, HPF with GI254023X or HPF with DAPT (*n* = 6 per group). The data were pooled from two independent experiments. **d** Quantification of cDC1-associated Toll-like receptor mRNA expression by qPCR in Fl-BMDCs after 6 h of treatment with PBS, PA0833, HPF, HPF with GI254023X or HPF with DAPT (*n* = 3). **e** Quantification of cDC2-associated Toll-like receptor mRNA expression by qPCR in Fl-BMDCs after 6 h of treatment with PBS, PA0833, HPF, HPF with GI254023X or HPF with DAPT (*n* = 3). **f** Quantification of *Irf4* or *Klf4* mRNA expression by qPCR in Fl-BMDCs after 6 h of treatment with PBS, PA0833, HPF, HPF with GI254023X or HPF with DAPT (*n* = 3). **g** Representative flow cytometry histogram (left) and quantification (right) of ESAM^+^ cell frequencies gated on CD11c^+^ MHCII^+^ SIRPa^+^ cells in Fl-BMDCs after 12 h of treatment with PBS, PA0833, HPF, HPF with GI254023X or HPF with DAPT (*n* = 6 per group). The data were pooled from two independent experiments. **h** Serum levels of IL-23 secreted from Fl-BMDCs after 12 h of treatment with PBS, PA0833, HPF, HPF with GI254023X or HPF with DAPT (*n* = 6 per group). The data were pooled from two independent experiments. Each data point indicates a biological replicate in (**a**, **c**, **g**, and **h**). Each data point indicates a biological replicate derived from the average of three technical replicates in (**b**,**d**, **e**, and **f**). The data are presented as the means ± s.e.m.s. Statistical significance was tested via one-way ANOVA followed by Tukey’s multiple comparisons test (**a**, **b**, **f**, **g** and **h**) and two-way ANOVA followed by Tukey’s multiple comparisons test (**c**–**e**)

To validate the role of ADAM10-Notch signaling in Hla_H35A_-mediated DC differentiation, we first assessed MHC-II expression in two distinct DC populations. Consistent with our previous findings, the inhibition of ADAM10 significantly impeded the Hla_H35A_ fusion-mediated upregulation of MHC-II (Fig. 3c). Treatment with DATP, a γ-secretase inhibitor that blocks S3 cleavage of the Notch receptor and inhibits the release of the NICD, led to a notable reduction in MHC-II expression in Fl-BMDCs (Fig. 5a and Supplementary Fig. 6a). Importantly, by knocking down the Notch1 and Notch2 receptors, we found that the Notch2 receptor may play a more significant role in ADAM10-Notch-mediated signaling (Supplementary Fig. 6b). Moreover, *Ccr7* expression was significantly increased upon Hla_H35A_ fusion in Fl-BMDCs, and this increase was markedly suppressed upon ADAM10 or NICD inhibition, suggesting that Hla_H35A_ fusion promotes DC migration to lymph nodes (Fig. 5b). Moreover, our studies revealed that PA0833 and HPF decreased the frequency of cDC1s (Fig. 5c). Conversely, both PA0833 and HPF promoted cDC2 differentiation, with the Hla_H35A_ fusion protein showing greater efficacy in an ADAM10/NICD-dependent manner (Fig. 5c). Among them, Notch2 was identified as the predominant regulator of cDC2 development (Supplementary Fig. 6c). NICD inhibition decreased the frequencies of both cDC1 and cDC2 (Fig. 5c), whereas the observed decrease in cDC1 frequency may be attributable to DAPT-induced cDC1 death (Supplementary Fig. 6d).

To determine whether the observed effect was caused by PA0833 or Hla_H35A_, we constructed fusion proteins of Hla_H35A_ with the antigens GlnH from *Klebsiella pneumoniae* and IsdB from *Staphylococcus aureus*,^19,20^ and the results revealed that the Hla_H35A_ fusion had a similar effect on different antigens (Supplementary Fig. 6e, f). In splenic DCs, Hla_H35A_ similarly promoted cDC2 differentiation while suppressing cDC1 development, accompanied by decreased pre-DC2 frequencies and increased pre-DC1 frequencies (Supplementary Fig. 6g–i). Neither individual antigens nor fusion antigens altered pre-DC3/DC3 proportions (Supplementary Fig. 6i), suggesting that DC3 differentiation may depend primarily on GM-CSF signaling.^21^ Furthermore, Hla_H35A_ fusion modulated TLR expression profiles during DC differentiation, increasing the expression of cDC2-associated TLRs (*Tlr1*, *Tlr2*, *Tlr5*, and *Tlr7*) while downregulating the expression of cDC1-associated TLRs (*Tlr3*, *Tlr11*, and *Tlr12*) (Fig. 5d, e).^22^ These changes were ADAM10/NICD dependent, as inhibition reversed the effects. Collectively, our findings demonstrate that the Hla_H35A_ fusion promotes cDC maturation via ADAM10-Notch signaling, preferentially driving cDC2 differentiation.

Owing to the heterogeneity of cDC2s, we analyzed key transcription factors regulating cDC2 subsets. PA0833 significantly increased *Irf4* expression, which was further enhanced by the Hla_H35A_ fusion in an ADAM10/NICD-dependent manner (Fig. 5f). Conversely, both PA0833 (~2.4-fold) and HPF (~4.5-fold) suppressed *Klf4* expression, with partial recovery following ADAM10/NICD inhibition (Fig. 5f), which is consistent with mutually exclusive Notch2/KLF4 expression defining cDC2 subsets.^14^ To assess Notch2-dependent cDC2 differentiation, we quantified ESAM^+^ cDC2s. Hla_H35A_ fusion significantly expanded this population (Fig. 5g), corroborating our RNA-seq data showing upregulation of the Notch2-associated gene *Dnase1l3* and downregulation of the KLF4-dependent genes *Mgl2* and *Clec10a* (Fig. 2a).^22,23^ ESAM^+^ cDC1s exhibited a similar expansion trend (Supplementary Fig. 7a), and ADAM10/NICD inhibition reduced the number of ESAM^+^ cells in both subsets (Fig. 5g, Supplementary Fig. 7a). The ESAM^+^ subtype specifically required ADAM10-Notch2 signaling (Supplementary Fig. 7b, c), a phenomenon absent in Mo-BMDCs (Supplementary Fig. 7d) but consistent across other fusion antigens (HGF/HIF; Supplementary Fig. 7e, f).

Previous studies have shown that Notch2-dependent cDCs in the gut contribute to IL-23 secretion.^24^ Compared with PA0833, the Hla_H35A_ fusion promoted the secretion of IL-23 by cDCs, and IL-23 levels were significantly reduced by GI254023X or DAPT treatment (Fig. 5h). These results establish that Hla_H35A_ fusion directs Notch2-dependent cDC2 lineage commitment via ADAM10-Notch signaling while suppressing KLF4-dependent cDC2 development.

### Hla_H35A_ mediates the optimal CD4 T-cell response via ADAM10-Notch signaling in cDCs

**Fig. 6:**
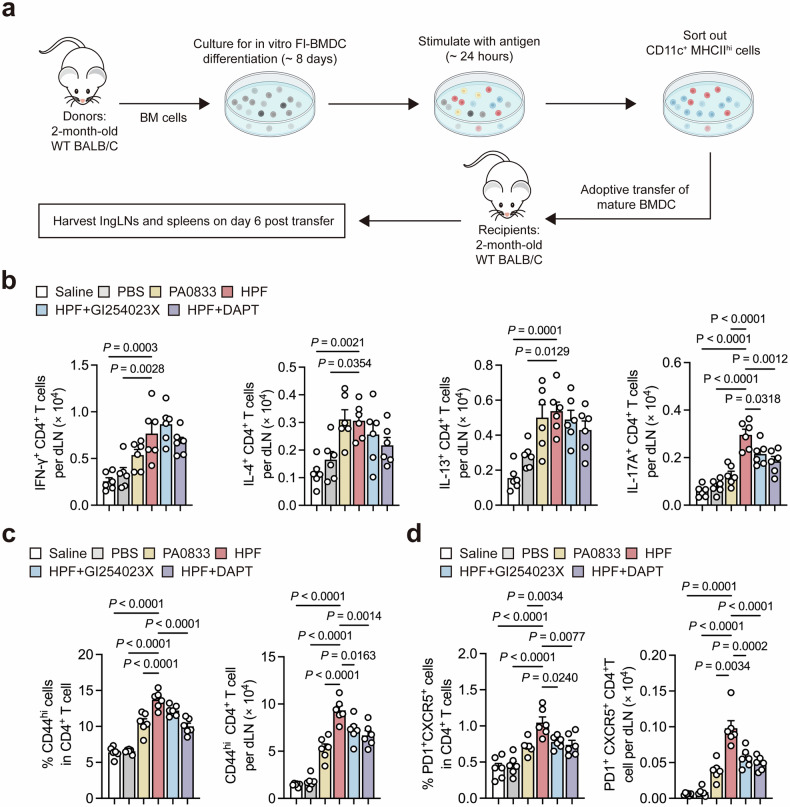
Hla_H35A_ promotes optimal Th17 and Tfh responses in dLNs by activating ADAM10-Notch signaling in cDCs. **a** Experimental design for the BMDC transfer model. Bone marrow (BM) cells from donor mice were cultured, stimulated and then transferred to recipient mice. The dLN and spleen of the recipient mice were analyzed on day 6 after transfer. Each mouse was intramuscularly injected with 5 × 10^5^ CD11c^+^MHCII^hi^ cells. **b** Numbers of cytokine-expressing CD4^+^ T cells in the dLNs on day 6 in mice that received Fl-BMDCs after 24 h of treatment with PBS, PA0833, HPF, HPF with GI254023X or HPF with DAPT (*n* = 6 per group). The data were pooled from two independent experiments. **c** Frequencies of CD44^hi^ CD4^+^ T cells (left) and numbers of CD44^hi^ CD4^+^ T cells (right) in the dLNs on day 6 in mice that received Fl-BMDCs after 24 h of treatment with PBS, PA0833, HPF, GI254023X or HPF with DAPT (*n* = 6 per group). The data were pooled from two independent experiments. **d** Frequencies of PD1^+^CXCR5^+^ cells among CD44^hi^ CD4^+^ T cells (left) and numbers of PD1^+^CXCR5^+^CD44^hi^ CD4^+^ T cells (right) in the dLNs on day 6 in mice that received Fl-BMDCs after 24 h of treatment with PBS, PA0833, HPF, GI254023X or HPF with DAPT (*n* = 6 per group). The data were pooled from two independent experiments. Each data point indicates a biological replicate in (**b**–**d**). The data are presented as the means ± s.e.m.s. Statistical significance was tested via one-way ANOVA followed by Tukey’s multiple comparisons test in (**b**–**d**). dLNs draining lymph nodes

To investigate Hla_H35A_-specific CD4⁺ T-cell priming by cDCs while excluding interference from other immune cells (e.g., pDCs), we adoptively transferred in vitro-primed CD11c^+^MHCII^hi^ Fl-BMDCs (treated with antigens/inhibitors) into recipient mice. CD4^+^ T-cell responses were analyzed 6 days post-transfer (Fig. 6a, Supplementary Fig. 8a). Hla_H35A_ fusion-primed cDCs selectively expanded IL-17A^+^ Th cells in dLNs without significantly increasing the number of IFN-γ^+^, IL-4^+^, or IL-13^+^ subsets (Fig. 6b). This finding aligns with the GO and KEGG results in Fl-BMDCs, where HPF induced more significant Th17 cells (~2.4-fold) than did PA0833 (Fig. 2c, d). This Th17 induction was abrogated by ADAM10/NICD inhibition. In contrast, T-cell responses in the spleen were not activated at 6 days after cDC transfer (Supplementary Fig. 8b), which is consistent with the CD44ʰⁱ CD4^+^ T-cell frequencies in the dLNs and spleen (Fig. 6c, Supplementary Fig. 8c). Thus, Hla_H35A_ fusion specifically enhances Th17 responses through ADAM10-Notch signaling in cDCs.

Given the established role of cDC2s, particularly ESAM⁺ subsets, in Tfh cell induction,^25^ we analyzed the frequency and number of PD1^+^CXCR5^+^CD44^hi^CD4^+^ T cells in dLNs. Compared with PA0833-primed DCs, Hla_H35A_ fusion-primed DCs significantly increased Tfh cell generation (Fig. 6d). This effect was ADAM10/NICD dependent, as inhibition of ADAM10/NICD reduced both the Tfh cell frequency and number (Fig. 6d). Notably, splenic Tfh cells were not affected by cDCs following either type of stimulation during this period (Supplementary Fig. 8d). Therefore, Hla_H35A_ fusion facilitates Tfh cell priming through ADAM10-Notch signaling in cDCs, which plays a crucial role in providing essential help to GC B cells in initiating the humoral immune response. These data demonstrate that Hla_H35A_ fusion efficiently induces Tfh cells through ADAM10-Notch signaling in cDCs.

### Hla_H35A_ improves vaccine efficacy by activating ADAM10-Notch signaling in cDCs in vivo

**Fig. 7:**
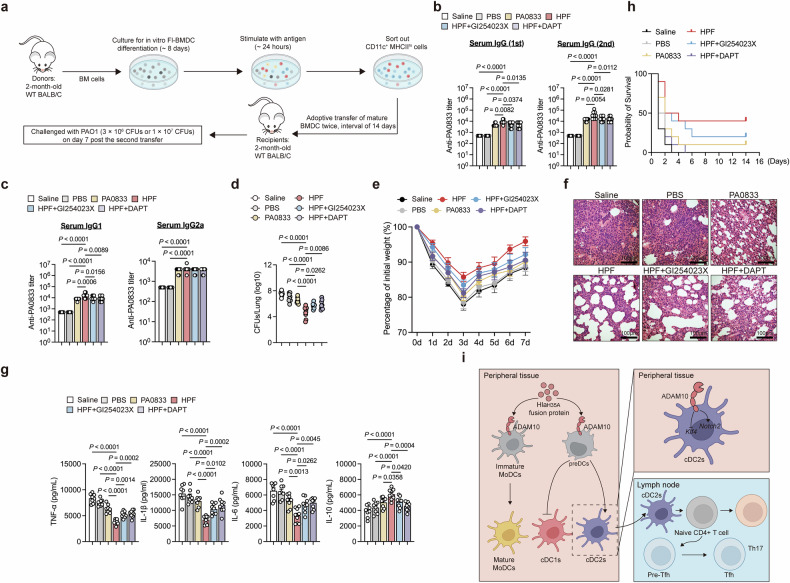
Hla_H35A_ improves the immunogenicity and protective efficacy of carried antigens by activating ADAM10-Notch signaling in cDCs. **a** Experimental design for the Fl-BMDC transfer model. Each mouse was intramuscularly injected with 5 × 10^5^ CD11c^+^MHCII^hi^ cells. **b** Serum PA0833-specific IgG levels in mice following two adoptive transfers of Fl-BMDCs treated for 24 h with PBS, PA0833, HPF, HPF with GI254023X, or HPF with DAPT (*n* = 8–9 per group). The data were pooled from two independent experiments. **c** Serum PA0833-specific IgG1 or IgG2a levels in mice following two adoptive transfers of Fl-BMDCs treated for 24 h with PBS, PA0833, HPF, HPF with GI254023X, or HPF with DAPT (*n* = 8–9 per group). The data were pooled from two independent experiments. **d** Bacterial loads in the lungs determined 48 h after challenge with a sublethal dose (3 × 10^6^ CFU) of PAO1 in mice following two adoptive transfers of Fl-BMDCs treated for 24 h with PBS, PA0833, HPF, HPF with GI254023X, or HPF with DAPT (*n* = 8–9 per group). The data were pooled from two independent experiments. **e** Body weight was measured 7 d after challenge with a sublethal dose (3 × 10^6^ CFU) of PAO1 in mice following two adoptive transfers of Fl-BMDCs treated for 24 h with PBS, PA0833, HPF, HPF with GI254023X, or HPF with DAPT (*n* = 7 per group). **f** Representative H&E staining of lung sections obtained 48 h after challenge with a sublethal dose (3 × 10^6^ CFU) of PAO1 from mice following two adoptive transfers of Fl-BMDCs treated for 24 h with PBS, PA0833, HPFs with GI254023X, or HPFs with DAPT (*n* = 3 per group). **g** TNF-α, IL-1β, IL-6, and IL-10 levels in the lungs were determined 48 h after challenge with a sublethal dose (3 × 10^6^ CFU) of PAO1 in mice following two adoptive transfers of Fl-BMDCs treated for 24 h with PBS, PA0833, HPF, GI254023X, or HPF with DAPT (*n* = 8–9 per group). The data were pooled from two independent experiments. **h** Kaplan–Meier survival curves of mice challenged with a lethal dose (1 × 10^7^ CFU) of PAO1, measured 14 days post-infection, following two adoptive transfers of Fl-BMDCs treated for 24 h with PBS, PA0833, HPF, HPF with GI254023X, or HPF with DAPT (*n* = 10 per group). **i** Schematic diagram of the proposed mechanism by which Hla_H35A_ enhances immunoprotection via ADAM10 on the surface of DCs. Each data point indicates a biological replicate in (**b**–**d** and **g**). The data are presented as the means ± s.e.m.s. Statistical significance was tested via one-way ANOVA followed by Tukey’s multiple comparisons test in (**b**–**d** and **g**)

To investigate whether Hla_H35A_ fusion protects mice against PAO1 infection by activating ADAM10-Notch signaling in cDCs in vivo, we employed an adoptive transfer model in which Fl-BMDCs were used (Fig. 7a). Compared with PA0833 controls, Hla_H35A_ fusion-stimulated cDCs significantly elevated PA0833-specific IgG titers, and this effect was attenuated by ADAM10/NICD inhibition (Fig. 7b). Moreover, the fusion antigen promoted a greater IgG1 response than did IgG2 (Fig. 7c), which was consistent with the direct immunization results (Fig. 1c). However, systemic inhibition of ADAM10 led to decreased IgG2a, but ADAM10/NICD suppression in cDCs did not have this effect (Figs. 1c, 7c). Overall, Hla_H35A_ fusion augments antigen replication-specific humoral immunity via ADAM10-Notch signaling in cDCs.

To evaluate vaccine-mediated protection, we assessed bacterial control in the adoptive transfer model. Compared with PA0833-primed cells, Hla_H35A_ fusion-stimulated cDCs demonstrated superior control of the PAO1 burden (Fig. 7d), an effect that was abrogated by ADAM10/NICD inhibition (Fig. 7d). Moreover, recipients of HPF-stimulated cDCs presented minimal weight loss (Fig. 7e) and reduced lung pathology (Fig. 7f). Lung cytokine profiling revealed decreased levels of TNF-α, IL-1β, and IL-6, along with elevated IL-10, in the HPF-stimulated cDC group (Fig. 7g). Following a lethal challenge, survival rates increased from 10% (PA0833) to 40% with HPF-stimulated cDCs but decreased to 15–20% after ADAM10/NICD inhibition (Fig. 7h). Thus, the Hla_H35A_ fusion enhances antibody titers and protection against bacterial infection in mice by activating ADAM10-Notch signaling in cDCs.

### Hla_H35A_ promotes human MoDC maturation and Notch activation via ADAM10

**Fig. 8:**
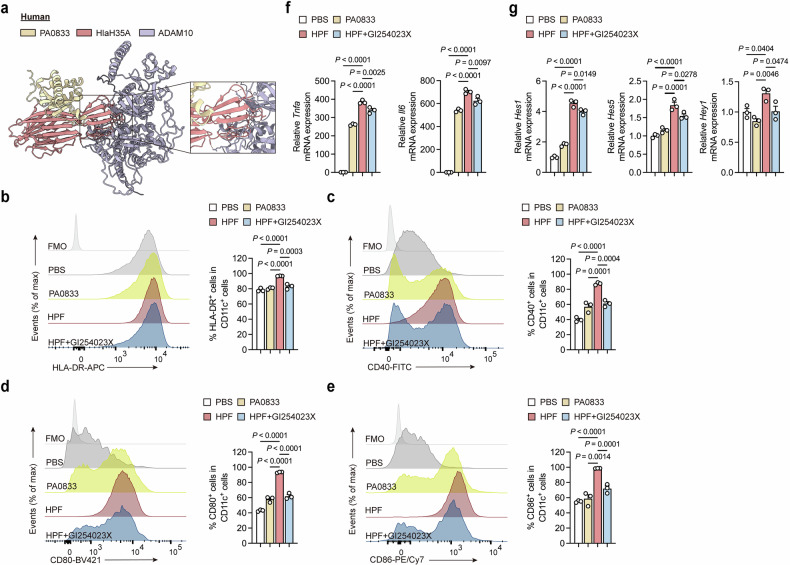
Hla_H35A_ induces human MoDC maturation via ADAM10. **a** Predicted model of the interaction of HPF with human ADAM10 (pLDDT = 73.6). The predicted local distance difference test score (pLDDT) was used to evaluate per-residue confidence, with values ≥ 70 indicating reliable backbone modeling. **b** Representative flow cytometry histogram (left) and quantification (right) of MHCII^+^ cell frequencies gated on CD11c^+^ cells from Fl-BMDCs after 6 h of treatment with PBS, PA0833, HPF, or HPF with GI254023X (*n* = 3 per group). **c** Representative flow cytometry histogram (left) and quantification (right) of CD40^+^ cell frequencies gated on CD11c^+^ cells from Fl-BMDCs after 6 h of treatment with PBS, PA0833, HPF, or HPF followed by GI254023X (*n* = 3 per group). **d** Representative flow cytometry histogram (left) and quantification (right) of CD80^+^ cell frequencies gated on CD11c^+^ cells from Fl-BMDCs after 6 h of treatment with PBS, PA0833, HPF, or HPF with GI254023X (*n* = 3 per group). **e** Representative flow cytometry histogram (left) and quantification (right) of CD86^+^ cell frequencies gated on CD11c^+^ cells from Fl-BMDCs after 6 h of treatment with PBS, PA0833, HPF, or HPF with GI254023X (*n* = 3 per group). **f** Quantification of *Tnfa* or *Il6* mRNA expression by qPCR in Fl-BMDCs after 6 h of treatment with PBS, PA0833, HPF, or HPF with GI254023X (*n* = 3 per group). **g** Quantification of *Hes1, Hes5*, or *Hey1* mRNA expression by qPCR in Fl-BMDCs after 6 h of treatment with PBS, PA0833, HPF, or HPF with GI254023X (*n* = 3 per group). Each data point indicates a biological replicate in (**b**–**e**). Each data point indicates a biological replicate derived from the average of three technical replicates in (**f** and**g**). The data are presented as the means ± s.e.m.s. Statistical significance was tested via one-way ANOVA followed by Tukey’s multiple comparisons test in (**b**–**g**)

To assess the conservation of Hla_H35A_ function in humans, we first predicted the interaction between HPF and human ADAM10 via AlphaFold2 (Fig. 8a). The result indicates that the interaction between Hla_H35A_ and ADAM10 is conserved across mice and humans. Next, we differentiated human peripheral blood mononuclear cells (PBMCs) into monocyte-derived dendritic cells (MoDCs) to investigate the functional role of Hla_H35A_ in human DCs. The viability of human MoDCs treated with PA0833, HPF, or HPF with GI254023X was maintained (Supplementary Fig. 9a). We subsequently examined the effect of Hla_H35A_ fusion on DC maturation (Supplementary Fig. 9b). Consistent with observations in mice, Hla_H35A_ fusion drove ADAM10-dependent MoDC maturation (Fig. 8b–e), accompanied by increased TNF-α/IL-6 transcription (Fig. 8f). Furthermore, Hla_H35A_ fusion increased the expression of Notch target genes (*Hes1*, *Hes5*, and *Hey1*) in an ADAM10-dependent manner (Fig. 8g, Supplementary Fig. 9c). These results demonstrate that the Hla_H35A_ fusion recapitulates the immunomodulatory functions of Hla_H35A_ in human MoDCs, which drive maturation and Notch activation via ADAM10, highlighting its potential as a human immune modulator.

## Discussion

Here, we aimed to elucidate how Hla_H35A_ enhances the immunogenicity and protective efficacy of fused antigens by targeting ADAM10. Our data revealed that Hla_H35A_ targets ADAM10 on myeloid cells, a step that is essential for subsequent antigen uptake and downstream signaling. Targeting ADAM10 enhances immune protection, as demonstrated by reduced vaccine efficacy, including decreased antibody titers and elevated bacterial loads following immunization with Hla_H35A_-fused antigens and ADAM10 inhibition. More importantly, ADAM10 blockade impairs development and function across immune cell populations, highlighting the importance of ADAM10 signaling in immune protection.^11^

APCs, particularly DCs, play crucial roles in connecting innate and adaptive immunity and are key targets for vaccines. DCs are distributed across all tissues in the human body and possess robust phagocytic capabilities in their immature state, enabling continuous surveillance of the surrounding environment.^26^ Most adjuvants and delivery systems aim to enhance T-cell responses via the classical three-signal paradigm: TCR engagement, costimulation, and cytokine production.^27^ Our study demonstrated that the Hla_H35A_ fusion augments antigen uptake in DCs, expanding our understanding of its role in myeloid APCs. Moreover, the ADAM10-Hla_H35A_ interaction significantly increased the ability of cDCs and Mo-DCs, which are crucial for bacterial infection, to induce T-cell priming signals and shape a moderate inflammatory environment, resulting in enhanced protective immunity. Importantly, neither the Hla_H35A_ fusion nor the antigen it carries affects the transcription of the ADAM10 receptor throughout this process. During this period, Hla_H35A_ does not induce inflammasome activation, avoiding premature cell death to ensure the transmission of information to the adaptive immune system.

However, the classical three-signal paradigm does not fully explain pathogen-specific DC programming, as diverse PRRs often elicit overlapping downstream effects.^28^ Consequently, DCs may acquire distinct phenotypes or functional states during pre-DC differentiation in response to microenvironmental cues, shaping subsequent immunity.^29^ Our work revealed that multiple protein antigens initially induce pre-DCs to differentiate into cDC2s while inhibiting their differentiation into cDC1s. Hla_H35A_ fusion further activates ADAM10-Notch signaling and enhances cDC2 differentiation across species, indicating functional conservation. Notably, short-term antigen exposure (<12 h) failed to drive the differentiation of pre-DC3s into DC3s, suggesting that GM-CSF rather than pathogen-derived signals may primarily govern this process.^21,30^ Critically, we discovered that the Hla_H35A_ fusion protein mainly promotes the maturation of cDCs rather than MoDCs through ADAM10-Notch signaling. While Notch1 and Notch2 play distinct roles in cDC function and ADAM10 can activate both Notch1 and Notch2, our results indicate that, at least in vitro, Hla_H35A_ fusion mainly shapes the function of cDCs, especially cDC2s, through ADAM10–Notch2 signaling. This effect is most likely attributable to the predominance of Notch2 receptors among the Notch receptors expressed on the surface of DCs.

Research has confirmed the presence of Notch2-dependent cDC2s in various tissues, including the spleen, lungs, and mesenteric lymph nodes.^29^ In vitro studies have shown that DL1-mediated Notch2 activation can drive the generation of Notch2-dependent cDC2s, especially ESAM^+^ cDC2s,^23^ demonstrating that Notch2-dependent cDC1/cDC2 development depends on local Notch signals within pre-cDC microenvironments. Consistently, our data confirmed that Hla_H35A_ fusion-induced cDC2s exhibit a Notch2-dependent cDC2 phenotype rather than a Klf4-dependent phenotype, as evidenced by the appearance of ESAM^+^ cDC2 subtypes, secretion of IL-23, and reduced expression of Klf4-dependent cDC2-related genes. We also observed a modest increase in ESAM⁺ cDC1s via ADAM10–Notch2 signaling. However, the overall cDC1 frequency reduction and low induction suggest that these cells likely contribute minimally to immune responses.

Notch2-dependent cDC2s play crucial roles in regulating type III immune responses against extracellular pathogens, such as the gut of *Citrobacter rodentium*.^24^ In our model, adoptively transferred Hla_H35A_-primed cDCs selectively induced Th17 (not Th2) responses in dLNs, indicating that Notch2-dependent cDC2s preferentially drive Th17 differentiation. Although mucosal immunization was not employed, which may induce robust mucosal immunity and that lung CD4 TRM cells are derived from Th17 cells, its synergistic effect with other T cells in the lymph nodes can still induce a significant immune protection effect.^31–33^ Furthermore, the Notch2-dependent cDC2 subset plays an irreplaceable role in promoting CD4⁺ Tfh cells and thereby supporting humoral immune responses.^25^ Our study also revealed that Hla_H35A_-induced cDC transfer resulted in increased Tfhs in the draining lymph nodes. This dual action results in elevated antigen-specific IgG1 titers and robust protection against acute lung infection, highlighting the potential of Hla_H35A_ as a novel adjuvant. Notably, we did not observe changes in splenic CD4^+^ T cells after the initial immunization with cDCs on day 6 post adoptive transfer, possibly because most of the cDC2s induced by Hla_H35A_ preferentially migrate to draining lymph nodes rather than being able to home to the spleen via the blood.^34,35^ Overall, our findings suggest that Hla_H35A_ could serve as a valuable carrier protein to induce the Th17 response and enhance Tfh function by targeting ADAM10 on cDCs, thereby improving the immune response to bacterial infections and offering a promising strategy for Th17-oriented vaccine design.

Furthermore, the intrinsic properties of Hla_H35A_, as a protein itself, enable its easy integration into composite adjuvant systems. For example, it can be effectively formulated with established adjuvants like aluminum hydroxide, which is widely recognized as a safe and effective vaccine adjuvant that enhances immunogenicity by improving antigen adsorption and stability.^36^ This compatibility allows Hla_H35A_ to synergize with external adjuvants to potentiate robust and tailored immune activation.^9^

Our study has several limitations, as we demonstrated that the Hla_H35A_ fusion protein primarily mediates the differentiation of cDC2s via the ADAM10-Notch2 pathway and enhances their functional capacity through in vitro assays and adoptive cell transfer models. However, given that both the Notch1 and Notch2 receptors are highly expressed on cDCs and that the ADAM10-Notch1 signaling axis has been shown to regulate type 2 immune responses and IgE production,^37^ it remains unclear whether this signaling pathway contributes to Hla_H35A_-induced protective immunity in vivo. Therefore, the use of ADAM10, Notch1, and Notch2 knockout mouse models could provide valuable insights into how antigens modulate cDC activity in tissues through the ADAM10-Notch signaling pathway. Moreover, although the Hla_H35A_ fusion antigen and PA0833 have no cytotoxic effects on MoDCs, potential adverse effects and broader safety concerns warrant further investigation.^38–40^

## Materials and methods

### Mice

Female BALB/c and C57BL/6 mice (8–10 weeks old) were procured from Beijing Charles River Laboratory Animal Technology Co., Ltd. All the mice were maintained in specific pathogen-free (SPF) animal facilities. The mice were euthanized following the guidelines set by the American Veterinary Medical Association for the Euthanasia of Animals. The study protocols were approved by the Institutional Animal Care and Use Committee (IACUC) of Third Military Medical University.

### Reagents

Recombinant mouse GM-CSF (Sino Biological, Cat# 51048-MNAH), recombinant mouse IL-4 (Sino Biological, Cat# 51084-MNAE), and recombinant human Flt3L (Sino Biological, Cat# 10315-HNAE) were utilized for the cultivation of bone marrow-derived dendritic cells (BMDCs). An ADAM10 inhibitor (GI254023X, Sellect, Cat# S8660) was used to inject mice intraperitoneally (100 mg/kg) or treat BMDCs (20 μM/1 × 10^6^ cells). The Notch inhibitor DAPT (Selleck, Cat# S2215) was used to treat BMDCs (20 μM/1 × 10^6^ cells). BMDCs were pretreated with GI254023X and DAPT (NICD inhibitor) for 24 h prior to downstream assays. Primary DCs were pretreated with GI254023X and DAPT for 16 h prior to downstream applications.

### Protein expression and purification

Recombinant PA0833, Hla_H35A_-PA0833 (HPF), GlnH, Hla_H35A_-GlnH (HGF), IsdB, and Hla_H35A_-IsdB (HIF) were produced via *E. coli* expression systems, as described previously.^9^ Briefly, codon-optimized genes encoding the desired proteins were cloned and inserted into an expression vector derived from the pGEX-6p-1 plasmid (Novagen) by Sheng Gong Biological Engineering (Shanghai, China). *E. coli* cells transformed with these expression plasmids were induced with 0.2 mM isopropyl-β-d-thiogalactopyranoside (IPTG) at 16 °C overnight and then harvested and homogenized via a nanohomogenization machine (ATS Engineering). The GST-tagged protein was captured with glutathione-Sepharose, followed by cleavage of the GST tag via PreScission Protease (GE Healthcare) after washing away nonspecific binding proteins. Lipopolysaccharide (LPS) contamination was further eliminated via ion-exchange chromatography, and residual endotoxin levels were quantified via the Limulus amebocyte lysate (LAL) assay (Xiamen Houshiji Biotech Co., Ltd.). The residual endotoxin level of the protein was <0.01 EU/μg. The protein purity was determined via SDS‒PAGE, and the concentration was determined via the BCA method.

### Antigen treatment

For in vitro experiments, cells (1 × 10^6^ per condition) were stimulated with 9.91 μg of PA0833, 30.15 μg of HPF, 15.00 μg of GlnH, 35.40 μg of HGF, 8.82 μg of IsdB or 29.41 μg of HIF in 50 μL of PBS. For in vivo experiments, the mice were immunized via intramuscular injection with 17.00 μg of PA0833 or 51.00 μg of HPF dissolved in 100 μL of saline.

### *P. aeruginosa* infection

The *P. aeruginosa* standard strain PAO1 was used for challenge in the acute pneumonia model. The concentration of *P. aeruginosa* was determined by measuring the absorbance at 600 nm. The bacteria were pelleted by centrifugation, washed twice with cold endotoxin-free PBS, and then resuspended to the desired density. For bacterial burden, histopathology, and cytokine analysis, the mice were infected with live PAO1 at a dose of 3 × 10^6^ colony-forming units (CFUs) via the intratracheal route and sacrificed at 48 h post infection. For body weight monitoring, the mice were infected with live PAO1 by administering 3 × 10^6^ CFUs via the intratracheal route and were observed for one week after infection. For survival assessment, the mice were infected with live PAO1 via intratracheally administered 1 × 10^7^ CFU of PAO1 and observed for 2 weeks post-infection.

### Antibody titers

The levels of PA0833-specific IgG, IgG1, and IgG2a in the serum of PA0833- or HPF-immunized mice were quantified via indirect ELISA. Briefly, 96-well high-binding microtiter plates were coated with 100 μL/well PA0833 (2 μg/mL in PBS) and incubated at 4 °C overnight. The plates were washed three times with 0.05% Tween-20 in PBS and blocked with 200 μL/well of PBS containing 1% BSA and 0.1% Tween-20 for 2 h at room temperature. Serial two-fold dilutions of serum samples (starting dilution, e.g., 1:1000) were prepared in assay diluent (PBS with 0.5% BSA, 0.05% Tween-20), and 100 μL of each dilution was added per well for 1 h at room temperature. After washing, bound antibodies were detected via the following HRP-conjugated secondary antibodies: goat anti-mouse IgG (1:1000; Beyotime, Cat# A0216), IgG1 (1:5000; Alpha Diagnostic International, Cat# 40126), or IgG2a (1:5000; Alpha Diagnostic International, Cat# 40127) in the same diluent for 1 h at room temperature. The plates were washed again and developed with 100 μL of TMB substrate (Beyotime, Cat# P0209) for 8 min, and the enzymatic reaction was stopped with 50 μL of stop solution (Beyotime, Cat# P0215). The absorbance was measured at 450 nm via a BioTek microplate reader. Endpoint Ig titers were defined as the highest serum dilution yielding an OD₄₅₀ > 2.1× background, following established ELISA criteria. Each sample was assayed in duplicate, and the OD readings were averaged for analysis.

### BMDC culture

BMDCs were cultured as described previously.^41,42^ Briefly, bone marrow cells collected from the femurs and tibias of BALB/c mice were cultured in RPMI-1640 medium (Gibco, Cat# C11875500BT) supplemented with 10% FBS (Moregate, Cat# 86827103), penicillin and streptomycin (Gibco, Cat# 15140122). For Flt3L‑driven cultures, the cells were incubated with 150 ng/mL human recombinant Flt3L without disturbance. Nonadherent Fl‑BMDCs were harvested on day 8, and ~90% CD11c⁺ purity was achieved. For the GM‑CSF/IL‑4 cultures, the bone marrow cells were cultured with 20 ng/mL recombinant mouse GM‑CSF and 10 ng/mL IL‑4, the medium was changed on day 3, and the Mo‑BMDCs were harvested on day 7, achieving approximately 70% CD11c⁺ purity.

### Human DC culture

Human monocyte-derived dendritic cells (Mo-DCs) were generated from peripheral blood mononuclear cells (PBMCs) obtained from NovoBiotechnology Co., Ltd. (Beijing, China). CD14^+^ monocytes were purified via the EasySep™ Human CD14 Positive Selection Kit (STEMCELL, Cat# 19359) and resuspended at 1 × 10^6^ cells/mL in RPMI‑1640 (Gibco) supplemented with 10% FBS (Moregate, Cat# 86827103), penicillin and streptomycin (Gibco, Cat# 15140122), recombinant human GM-CSF (100 ng/ml) and human IL-4 (100 ng/ml; both from R&D). The medium was changed on day 3, and MoDCs were harvested on day 6, resulting in >70% CD11c⁺ expression.

### Primary DC isolation

Primary DCs from all splenic DC subsets were isolated from C57BL/6 mouse spleens via enzymatic digestion followed by sequential immunomagnetic separation. Spleens were excised, minced, and digested at 37 °C for 30 min in RPMI-1640 medium supplemented with 5% FBS, 1 mg/mL collagenase D (Sigma-Aldrich, Cat# C5138-1G) and 20 µg/mL DNase I (Roche, Cat# 10104159001). The resulting cell suspensions were filtered through a 70 µm cell strainer and washed in PBS supplemented with 2% FBS to obtain single-cell suspensions. Given that the splenic pre-DC1, pre-DC2, DC1, DC2, and DC3 subsets express CD11c, while pro-DC3 cells lack CD11c but are positive for CD11b and Ly6C,^30^ the CD11c⁺ and CD11b⁺Ly6G⁻Ly6C⁺ cell populations were isolated separately and subsequently combined at a 1:1 ratio to ensure the inclusion of all the dendritic cell types. CD11c⁺ cells were positively selected via the EasySep™ Mouse CD11c Positive Selection Kit II (STEMCELL, Cat#18780), achieving >90% purity, and CD11b^+^Ly6G^−^Ly6C^hi/lo^ cells were enriched via negative selection via the EasySep™ Mouse Monocyte Isolation Kit (STEMCELL, Cat#19861), yielding approximately 92% monocyte purity. The two purified fractions were washed, counted, and combined in equal proportions before being resuspended in PBS with 2% FBS for downstream applications.

### RNAi and transfection

For siRNA-mediated knockdown of ADAM10, Notch1, or Notch2, mouse ADAM10 siRNA (siADAM10), mouse Notch1 siRNA (siNotch1), mouse Notch2 siRNA (siNotch2), and a nontargeting siRNA negative control (siNC) were synthesized by GENEWIZ (Suzhou, China). The sequences that specifically target mouse ADAM10, mouse Notch1 and mouse Notch2 are listed in Supplementary S1. For siRNA transfection, 2 × 10^5^ Fl-BMDCs were cultured in 6-well plates. A total of 100 μL of RPMI-1640 medium (Gibco, Cat# C11875500BT) was mixed with 2.5 μL of siRNA (20 μM) and 12 μL of HiPerFect Transfection Reagent (Qiagen, Cat# 301705) per well and added to the cultured cells. The cells were incubated for 48 h prior to downstream applications.

### Adoptive transfer

BMDCs were harvested 8 days after BALB/c mouse bone marrow cells were induced with Flt3L. Fl-BMDCs were collected after 24 h of different treatments. CD11c^+^MHCII^hi^ DCs were sorted from these cells with a FACSAria III flow cytometer. A total of 5 × 10^5^ cells in 200 μl of saline were administered via intramuscular injection into recipient mice. T cells in the LN or spleen were analyzed 6 days later.

### Cytokine detection

All cytokines were detected via ELISA. For in vitro experiments, supernatants were collected from cells (1 × 10^6^ cells/well). For in vivo experiments, supernatants were collected by homogenizing each mouse lung in PBS. All the supernatants were stored at −20 °C. Cytokines, including TNF-α (Thermo Fisher, Cat# 88-7324), IL-1β (Thermo Fisher, Cat# 88-7013 A), IL-6 (Thermo Fisher, Cat# 88-7064), IL-10 (Thermo Fisher, Cat# 88-7105), and IL-23 (Thermo Fisher, Cat# 88-7230), were assayed via the Mouse Uncoated ELISA Kit according to the manufacturer’s instructions.

### Tissue sampling and processing

The draining lymph nodes were gently dissociated via the plunger of a syringe, filtered through a 70 µm strainer, and then washed in PBS with 2% FBS. Spleens were mechanically disrupted, filtered similarly, and treated with cold 1× RBC lysis buffer on ice for 5 min, followed by washing in PBS with 2% FBS. Skeletal muscle at the intramuscular injection site (<3 g) was finely minced and digested in RPMI 1640 containing 5% FBS, 0.25 mg/mL Liberase (Roche, Cat# 5401020001), and 0.5 mg/mL DNase I (Roche, Cat# 10104159001) at 37 °C in a shaking incubator (15 rpm) for 90 min. Digestion was terminated by mechanical disruption, the samples were filtered through a 70 µm strainer and washed in PBS with 2% FBS. Digestion was terminated by mechanical disruption, and the mixture was filtered through 70 µm strainers, followed by washing with PBS supplemented with 2% FBS. All the cell suspensions were centrifuged, resuspended, counted, and filtered again through a 70 µm strainer to ensure that a single-cell suspension was suitable in PBS with 2% FBS for downstream applications.

### Flow cytometry

The cells (1 × 10⁶) were incubated with anti-CD16/32 (clone 2.4G2, STARTER, Cat# S0B0599-200 T) for 20 min at 4 °C to block Fc receptors and then stained with antibody cocktails in PBS containing 2% FBS (100 µL total volume) for 30 min at 4 °C. Dead cells were excluded via Fixable Viability Stain 700 (BD Biosciences, Cat# 564997) or Fixable Viability Dye eFluor 780 (eBioscience, Cat# 65-0865-18) staining. For intracellular cytokine detection, single-cell suspensions from murine draining lymph nodes and spleens were stimulated at 37 °C for 5 h with 50 ng/mL phorbol 12-myristate 13-acetate (Sigma-Aldrich, Cat# P8139-1MG), 1 µg/mL ionomycin (Sigma-Aldrich, Cat# I3909-1 ML), and 1 µg/mL GolgiStop (BD Pharmingen, Cat# 554724) to inhibit protein secretion. Following surface staining and viability labeling, the cells were fixed and permeabilized with the Foxp3 staining buffer set (eBioscience, Cat# 00-5523-00) and stained with antibodies of relevant specificities. The antibodies used to stain immune cells are listed in Table S2.

### RT‒qPCR

Total RNA was extracted with an EZ-10 total RNA extraction kit (Sangon Biotech, Cat# B618583-0100) according to the manufacturer’s instructions. cDNA was synthesized via reverse transcription via the PrimeScript RT Reagent Kit (Takara, Cat# RR037A). PCRs were performed via the QuantiNova SYBR PCR Mix Kit (Qiagen, Cat# 1129280). The primer sequences are listed in Table S3.

### Western blotting

Whole-cell lysates (WCLs) were prepared from 1 × 10⁶ cells via RIPA lysis buffer (BIOMIKY, Cat# MK035A) and incubated on ice for 30 min. The lysates were then centrifuged at 12,000 rpm for 15 min at 4 °C, and the supernatants were collected. Subsequently, 5× loading buffer was added, and the mixture was boiled at 100 °C for 10 min to denature the proteins. Protein samples were then separated on 10% Tris‒glycine gels (BIOMIKY, Cat# MK303) and transferred to nitrocellulose membranes (Merck Millipore, Cat# ISEQ00010). The membranes were blocked with 5% nonfat milk in TBST (0.1% Tween-20 in TBS) for 1 h at room temperature and incubated overnight at 4 °C with primary antibodies against N1ICD (1:1000, CST, Cat# 8216S), N2ICD (1:1000, CST, Cat# 4970T), β-actin (1:1000, CST, Cat# 5732T), Notch3 (1:1000, Santa Cruz, Cat# sc-515825), or Notch4 (1:1000, Santa Cruz, Cat# sc-393893). After three washes with TBST, the membranes were incubated with HRP-conjugated secondary antibodies—goat anti-rabbit IgG (1:1000, Beyotime, Cat# A0208) or goat anti-mouse IgG (1:1000, Beyotime, Cat# A0216)—for 2 h at room temperature. The protein bands were visualized via the ChemiDoc MP Imaging System (Bio-Rad).

### Protein interaction prediction

The 3D molecular structure of the proteins was predicted via AlphaFold2 and visualized via ChimeraX software.^43–45^ Protein interactions were predicted via AlphaFold Multimer. The predicted local distance difference test score (pLDDT) was used to evaluate per-residue confidence, with values ≥ 70 indicating reliable backbone modeling.

### Immunofluorescence staining

PA0833 and HPF were labeled with Alpha Fluor 488 NHS Ester (AAT Bioquest, Cat# 2815-1 ml) according to the manufacturer’s protocol.^46^ The cells were subsequently treated with the indicated concentrations of Alpha Fluor 488-labeled PA0833 and HPF. Then, the cells were fixed with 4% paraformaldehyde and permeabilized with PBS containing 0.3% Triton X-100. Anti-ADAM10 antibody (1:250, Santa Cruz, Cat# sc-48400) was then added to the cells, which were then incubated overnight at 4 °C. Finally, a goat anti-mouse (1:1000, Bioss, Cat# bs-0296G-AF647) secondary antibody was applied for 60 min at room temperature, and the cell nuclei were stained with DAPI (Bioss, Cat# C02-04002). Images were acquired via a laser scanning confocal microscope (Zeiss).

### CCK-8 assay

A CCK-8 kit (Biosharp, Cat# CCK8-500T) was used to evaluate cell proliferation activity according to the experimental procedure instructions. The cells were seeded into 96-well plates at a density of 2 × 10^4^ cells/well. Cell viability was measured at 24 h after incubation. The CCK8 mixture was added to 96-well plates (10 μL per well) and incubated at 37 °C for approximately 1.5 h.

### Hematoxylin and eosin (H&E) staining

Lungs were fixed in 10% formalin, embedded in paraffin, and sectioned into 4-μm sections. Subsequently, the lung sections were stained with H&E. All staining procedures were performed via the histology core at Southwest Hospital. Briefly, tissue sections were immersed in Harris hematoxylin for 10 s and then washed with tap water. The cleared sections were then reimmersed with eosin for approximately 30 s. The sections were washed with tap water until clear and then dehydrated in ascending alcohol solutions (50%, 70%, 80%, 95% × 2, 100% × 2). Afterwards, xylene was used to clear the sections three to four times. Finally, the sections were mounted on glass slides using Permount organic mounting medium for visualization.

### Bulk RNA sequencing

Total RNA was extracted from BMDCs isolated from 12 wild-type (WT) mice subjected to different treatments. For each sample, 5 × 10^6^ cells were collected, and RNA was extracted via the TRNzol Universal RNA Extraction Kit (TianGen, Cat# DP424) and then stored at –80 °C until further use. RNA libraries were prepared and sequenced on the DNBSEQ platform (BGI, Shenzhen, China) by Sangon Biotech Co., Ltd. Clean reads were mapped and quantified via StringTie to obtain transcript-level TPM values,^47^ and differential gene expression analysis was performed in R (v3.5.1) via DEGseq (v3.22.5),^48^ following the recommended workflow for experiments with biological replicates. Differentially expressed genes (DEGs) were defined as those with a *P* value < 0.05 and |log₂-fold change|>1 and were merged to construct a comprehensive TPM expression matrix. Downstream pathway enrichment analysis of DEGs was conducted via the Gene Ontology (GO) and Kyoto Encyclopedia of Genes and Genomes (KEGG) databases. Enriched pathways were filtered and displayed when *P* < 0.005 and Q < 0.1 for significance filtering.

### Statistical analysis

All the statistical analyses were performed via GraphPad Prism 9 (GraphPad Software). The data are presented as the means ± standard errors of the means (s.e.m.). The sample size or number of independent replicates is specified in the figure legend. Comparisons among three or more groups were performed via one-way or two-way ANOVA followed by Tukey’s multiple comparisons test, as appropriate. Survival data were analyzed via the log-rank test. Differences were considered statistically significant at *P* values < 0.05.

## Supplementary information

SUPPLEMENTAL MATERIAL

## Data Availability

The transcriptomic sequencing data are available at Gene Expression Omnibus (GEO) under accession no. GSE307316. All other data associated with this study are presented in the manuscript or the supplementary information.

## References

[1] Berube, B. J. & Bubeck Wardenburg, J. *Staphylococcus aureus* alpha-toxin: nearly a century of intrigue. *Toxins* **5**, 1140–1166 (2013). 23888516 10.3390/toxins5061140 PMC3717774

[2] Bischofberger, M., Iacovache, I. & van der Goot, F. G. Pathogenic pore-forming proteins: function and host response. *Cell Host Microbe* **12**, 266–275 (2012). 22980324 10.1016/j.chom.2012.08.005

[3] Wilke, G. A. & Bubeck Wardenburg, J. Role of a disintegrin and metalloprotease 10 in *Staphylococcus aureus* alpha-hemolysin-mediated cellular injury. *Proc. Natl Acad. Sci. USA* **107**, 13473–13478 (2010). 20624979 10.1073/pnas.1001815107 PMC2922128

[4] Caldera, J. R. et al. The characteristics of pre-existing humoral imprint determine efficacy of S. aureus vaccines and support alternative vaccine approaches. *Cell Rep. Med.* **5**, 101360 (2024). 38232694 10.1016/j.xcrm.2023.101360 PMC10829788

[5] Tsai, C. M. et al. Non-protective immune imprint underlies failure of *Staphylococcus aureus* IsdB vaccine. *Cell Host Microbe* **30**, 1163–1172 e1166 (2022). 35803276 10.1016/j.chom.2022.06.006 PMC9378590

[6] Karauzum, H. et al. Vaccine display on artificial bacterial spores enhances protective efficacy against *Staphylococcus aureus* infection. *FEMS. Microbiol. Lett*. **365**, fny190 (2018). 10.1093/femsle/fny190 PMC6454432 30084923

[7] Adhikari, R. P., Thompson, C. D., Aman, M. J. & Lee, J. C. Protective efficacy of a novel alpha hemolysin subunit vaccine (AT62) against *Staphylococcus aureus* skin and soft tissue infections. *Vaccine* **34**, 6402–6407 (2016). 27847174 10.1016/j.vaccine.2016.09.061 PMC5130608

[8] Liang, X., Yan, M. & Ji, Y. The H35A mutated alpha-toxin interferes with cytotoxicity of staphylococcal alpha-toxin. *Infect. Immun.* **77**, 977–983 (2009). 19103771 10.1128/IAI.00920-08 PMC2643624

[9] Zou, J. T. et al. Pore-forming alpha-hemolysin efficiently improves the immunogenicity and protective efficacy of protein antigens. *PLoS. Pathog.* **17**, e1009752 (2021). 34288976 10.1371/journal.ppat.1009752 PMC8294524

[10] Edwards, D. R., Handsley, M. M. & Pennington, C. J. The ADAM metalloproteinases. *Mol. Asp. Med.* **29**, 258–289 (2008). 10.1016/j.mam.2008.08.001 PMC7112278 18762209

[11] Lambrecht, B. N., Vanderkerken, M. & Hammad, H. The emerging role of ADAM metalloproteinases in immunity. *Nat. Rev. Immunol.* **18**, 745–758 (2018). 30242265 10.1038/s41577-018-0068-5

[12] Sprinzak, D. & Blacklow, S. C. Biophysics of Notch signaling. *Annu. Rev. Biophys.* **50**, 157–189 (2021). 33534608 10.1146/annurev-biophys-101920-082204 PMC8105286

[13] Nutt, S. L. & Chopin, M. Transcriptional networks driving dendritic cell differentiation and function. *Immunity* **52**, 942–956 (2020). 32553180 10.1016/j.immuni.2020.05.005

[14] Yin, X., Chen, S. & Eisenbarth, S. C. Dendritic cell regulation of T helper cells. *Annu. Rev. Immunol.* **39**, 759–790 (2021). 33710920 10.1146/annurev-immunol-101819-025146

[15] Zupancic, E. et al. Rational design of nanoparticles towards targeting antigen-presenting cells and improved T cell priming. *J. Control. Release* **258**, 182–195 (2017). 28511928 10.1016/j.jconrel.2017.05.014

[16] Silva, M. et al. A particulate saponin/TLR agonist vaccine adjuvant alters lymph flow and modulates adaptive immunity. *Sci. Immunol.* **6**, eabf1152 (2021). 34860581 10.1126/sciimmunol.abf1152 PMC8763571

[17] Craven, R. R. et al. *Staphylococcus aureus* alpha-hemolysin activates the NLRP3-inflammasome in human and mouse monocytic cells. *PLoS. ONE* **4**, e7446 (2009). 19826485 10.1371/journal.pone.0007446 PMC2758589

[18] Chow, K. V., Lew, A. M., Sutherland, R. M. & Zhan, Y. Monocyte-derived dendritic cells promote Th polarization, whereas conventional dendritic cells promote Th proliferation. *J. Immunol.* **196**, 624–636 (2016). 26663720 10.4049/jimmunol.1501202

[19] Chen, Z. et al. Vaccination with a trivalent *Klebsiella pneumoniae* vaccine confers protection in a murine model of pneumonia. *Vaccine* **42**, 126217 (2024). 39163713 10.1016/j.vaccine.2024.126217

[20] Chen, Z. et al. Immunodominance of epitopes and protective efficacy of HI antigen are differentially altered using different adjuvants in a mouse model of *Staphylococcus aureus* bacteremia. *Front. Immunol.* **12**, 684823 (2021). 34122448 10.3389/fimmu.2021.684823 PMC8190387

[21] Bourdely, P. et al. Transcriptional and functional analysis of CD1c(+) human dendritic cells identifies a CD163(+) subset priming CD8(+)CD103(+) T cells. *Immunity* **53**, 335–352 e338 (2020). 32610077 10.1016/j.immuni.2020.06.002 PMC7445430

[22] Brown, C. C. et al. Transcriptional basis of mouse and human dendritic cell heterogeneity. *Cell* **179**, 846–863.e824 (2019). 31668803 10.1016/j.cell.2019.09.035 PMC6838684

[23] Kirkling, M. E. et al. Notch signaling facilitates in vitro generation of cross-presenting classical dendritic cells. *Cell Rep.* **23**, 3658–3672 e3656 (2018). 29925006 10.1016/j.celrep.2018.05.068 PMC6063084

[24] Satpathy, A. T. et al. Notch2-dependent classical dendritic cells orchestrate intestinal immunity to attaching-and-effacing bacterial pathogens. *Nat. Immunol.* **14**, 937–948 (2013). 23913046 10.1038/ni.2679 PMC3788683

[25] Briseno, C. G. et al. Notch2-dependent DC2s mediate splenic germinal center responses. *Proc. Natl Acad. Sci. USA* **115**, 10726–10731 (2018). 30279176 10.1073/pnas.1809925115 PMC6196531

[26] Sallusto, F., Cella, M., Danieli, C. & Lanzavecchia, A. Dendritic cells use macropinocytosis and the mannose receptor to concentrate macromolecules in the major histocompatibility complex class II compartment: downregulation by cytokines and bacterial products. *J. Exp. Med.* **182**, 389–400 (1995). 7629501 10.1084/jem.182.2.389 PMC2192110

[27] Warrick, K. A., Vallez, C. N., Meibers, H. E. & Pasare, C. Bidirectional communication between the innate and adaptive immune systems. *Annu. Rev. Immunol.* **43**, 489–514 (2025). 40279312 10.1146/annurev-immunol-083122-040624 PMC12120936

[28] Jain, A. & Pasare, C. Innate control of adaptive immunity: beyond the three-signal paradigm. *J. Immunol.* **198**, 3791–3800 (2017). 28483987 10.4049/jimmunol.1602000 PMC5442885

[29] Minutti, C. M. et al. Distinct ontogenetic lineages dictate cDC2 heterogeneity. *Nat. Immunol.* **25**, 448–461 (2024). 38351322 10.1038/s41590-024-01745-9 PMC10907303

[30] Liu, Z. et al. Dendritic cell type 3 arises from Ly6C(+) monocyte-dendritic cell progenitors. *Immunity* **56**, 1761–1777 e1766 (2023). 37506694 10.1016/j.immuni.2023.07.001

[31] Kunzli, M. & Masopust, D. CD4(+) T cell memory. *Nat. Immunol.* **24**, 903–914 (2023). 37156885 10.1038/s41590-023-01510-4 PMC10343737

[32] Iwanaga, N. et al. Vaccine-driven lung TRM cells provide immunity against *Klebsiella* via fibroblast IL-17R signaling. *Sci. Immunol.* **6**, eabf1198 (2021). 34516780 10.1126/sciimmunol.abf1198 PMC8796208

[33] Amezcua Vesely, M. C. et al. Effector T(H)17 cells give rise to long-lived T(RM) cells that are essential for an immediate response against bacterial infection. *Cell* **178**, 1176–1188.e1115 (2019). 31442406 10.1016/j.cell.2019.07.032 PMC7057720

[34] Durai, V. & Murphy, K. M. Functions of murine dendritic cells. *Immunity* **45**, 719–736 (2016). 27760337 10.1016/j.immuni.2016.10.010 PMC5145312

[35] Mebius, R. E. & Kraal, G. Structure and function of the spleen. *Nat. Rev. Immunol.* **5**, 606–616 (2005). 16056254 10.1038/nri1669

[36] Shi, S. et al. Vaccine adjuvants: understanding the structure and mechanism of adjuvanticity. *Vaccine* **37**, 3167–3178 (2019). 31047671 10.1016/j.vaccine.2019.04.055

[37] Damle, S. R. et al. ADAM10 and Notch1 on murine dendritic cells control the development of type 2 immunity and IgE production. *Allergy* **73**, 125–136 (2018). 28745029 10.1111/all.13261 PMC5739941

[38] Gupta, S. & Pellett, S. Recent developments in vaccine design: from live vaccines to recombinant toxin vaccines. *Toxins* **15**, 563 (2023). 10.3390/toxins15090563 PMC10536331 37755989

[39] Findlow, H. & Borrow, R. Interactions of conjugate vaccines and co-administered vaccines. *Hum. Vaccin. Immunother.* **12**, 226–230 (2016). 26619353 10.1080/21645515.2015.1091908 PMC4962715

[40] Knuf, M., Kowalzik, F. & Kieninger, D. Comparative effects of carrier proteins on vaccine-induced immune response. *Vaccine* **29**, 4881–4890 (2011). 21549783 10.1016/j.vaccine.2011.04.053

[41] Brasel, K., De Smedt, T., Smith, J. L. & Maliszewski, C. R. Generation of murine dendritic cells from flt3-ligand-supplemented bone marrow cultures. *Blood* **96**, 3029–3039 (2000). 11049981

[42] Lutz, M. B. et al. Immature dendritic cells generated with low doses of GM-CSF in the absence of IL-4 are maturation resistant and prolong allograft survival in vivo. *Eur. J. Immunol.* **30**, 1813–1822 (2000). 10940870 10.1002/1521-4141(200007)30:7<1813::AID-IMMU1813>3.0.CO;2-8

[43] Meng, E. C. et al. UCSF ChimeraX: tools for structure building and analysis. *Protein Sci.* **32**, e4792 (2023). 37774136 10.1002/pro.4792 PMC10588335

[44] Mirdita, M. et al. ColabFold: making protein folding accessible to all. *Nat. Methods* **19**, 679–682 (2022). 35637307 10.1038/s41592-022-01488-1 PMC9184281

[45] Jumper, J. et al. Highly accurate protein structure prediction with AlphaFold. *Nature* **596**, 583–589 (2021). 34265844 10.1038/s41586-021-03819-2 PMC8371605

[46] Liang, F. et al. Vaccine priming is restricted to draining lymph nodes and controlled by adjuvant-mediated antigen uptake. *Sci. Transl. Med*. **9**, eaal2094 (2017). 10.1126/scitranslmed.aal2094 28592561

[47] Pertea, M. et al. StringTie enables improved reconstruction of a transcriptome from RNA-seq reads. *Nat. Biotechnol.* **33**, 290–295 (2015). 25690850 10.1038/nbt.3122 PMC4643835

[48] Wang, L. et al. DEGseq: an R package for identifying differentially expressed genes from RNA-seq data. *Bioinformatics* **26**, 136–138 (2010). 19855105 10.1093/bioinformatics/btp612

