# Supplementary material for: Alpha hemolysin enhances the immune response by modulating dendritic cell differentiation via ADAM10-Notch signaling
Source: Signal Transduct Target Ther. 2025 Oct 8;10:334. doi: 10.1038/s41392-025-02432-3 (PMC12508477; doi:10.1038/s41392-025-02432-3)
Supplement: Supplementary file 1 — SUPPLEMENTAL MATERIAL [file 41392_2025_2432_MOESM1_ESM.docx]

Supplementary Materials for

Alpha hemolysin enhances the immune response by modulating dendritic cell differentiation via ADAM10-Notch signaling

Ke Wang^1, 2, 3, 4^, Jingwen Liao^1^, Yue Yuan^1^, Zhifu Chen^1^, Qiang Gou^1^, Haiming Jing^1^, Mengmeng Liang^5,6^, Yuanda Tang^1^, Pengju Yan^1^, Xiaoqian Yu^1^, Zhuo Zhao^1^, Tianjun Sun^1^, Zhenping Xia^1^, Ting Yu^1,7^, Yaling Liao^1^, Hao Zeng^1^, Xiaoli Zhang^8*^, Quanming Zou^1*^, Jinyong Zhang^1*^

*Corresponding author: Xiaoli Zhang (xlzhang1981@126.com), Quanming Zou (qmzou2007@163.com), Jinyong Zhang (zhangjy198217@126.com)

**This file includes:**

Figures. S1 to S9

Tables S1 to S3

**
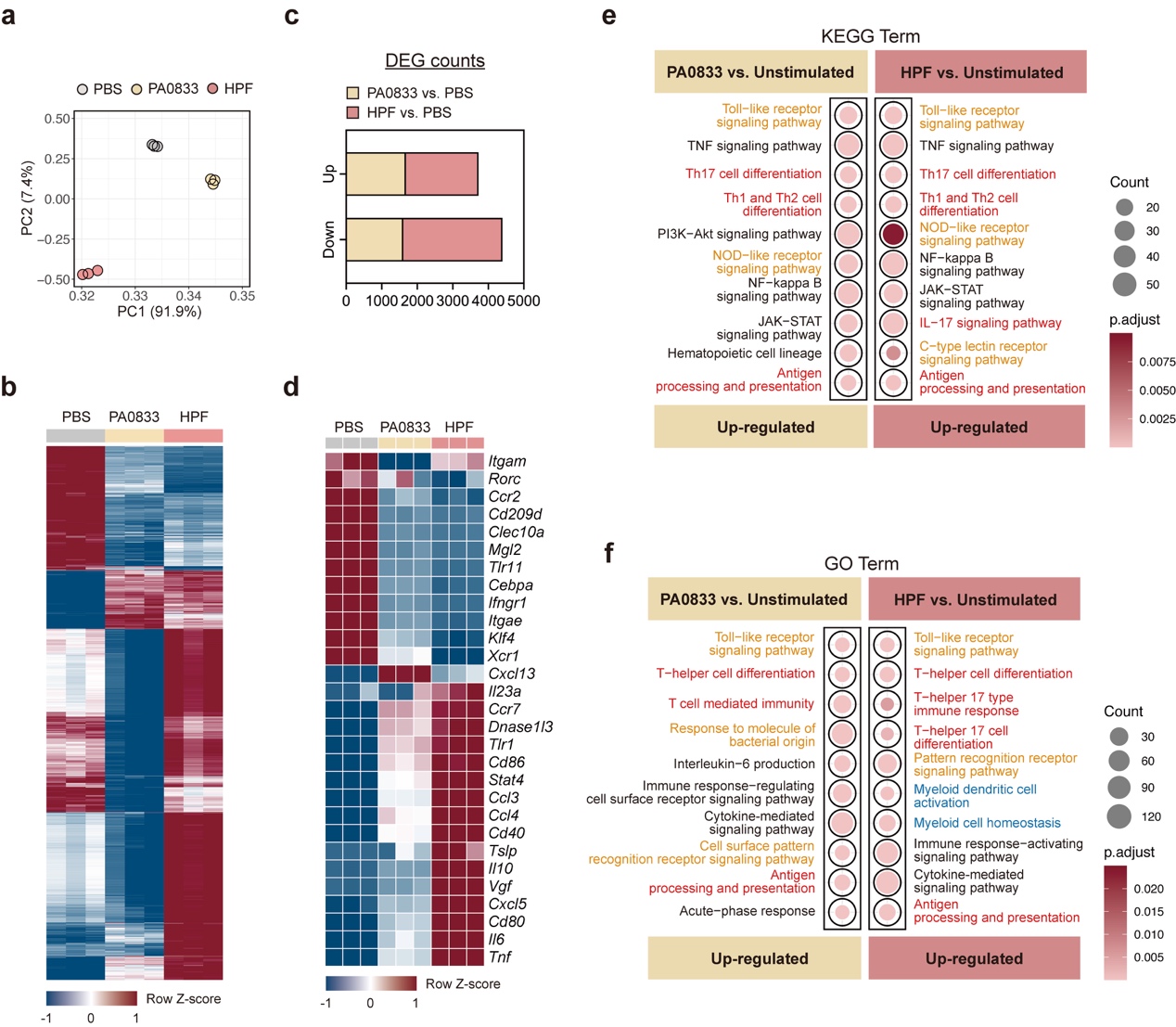
**

**Figure. S1. Bulk RNA-seq analysis of Hla_H35A_ fusion or antigen-treated Fl-BMDCs compared with unstimulated Fl-BMDCs.**

**a** PCA revealed a two-dimensional representation of the transcriptome profiles of Fl-BMDCs after 7.5 h of treatment with PBS, PA0833, or HPF (*n* = 3 per condition). **b** Heatmap of DEGs in Fl-BMDCs after 7.5 h of treatment with PBS, PA0833, or HPF. The relative expression abundance (rowwise Z score of the log2(TPM + 1), where TPMs denote transcripts per million; color scale) of genes (rows) across conditions (columns) is shown. **c** DEG counts between PA0833-stimulated and unstimulated or stimulated and unstimulated samples. **d** Heatmap of representative gene expression in Fl-BMDCs after 7.5 h of treatment with PBS, PA0833, or HPF. **e** KEGG analysis of upregulated genes in Fl-BMDCs after 7.5 h of treatment with PA0833 vs. unstimulated or HPF vs. unstimulated. Red indicates T-cell response-related signals, and orange indicates PRR-related signals. **f** GO enrichment analysis of upregulated genes in Fl-BMDCs treated with PA0833 vs. unstimulated or HPF vs. unstimulated. Red indicates T-cell response-related signals, blue indicates dendritic cell development-related signals, and orange indicates PRR-related signals. PCA: Principal component analysis.

**
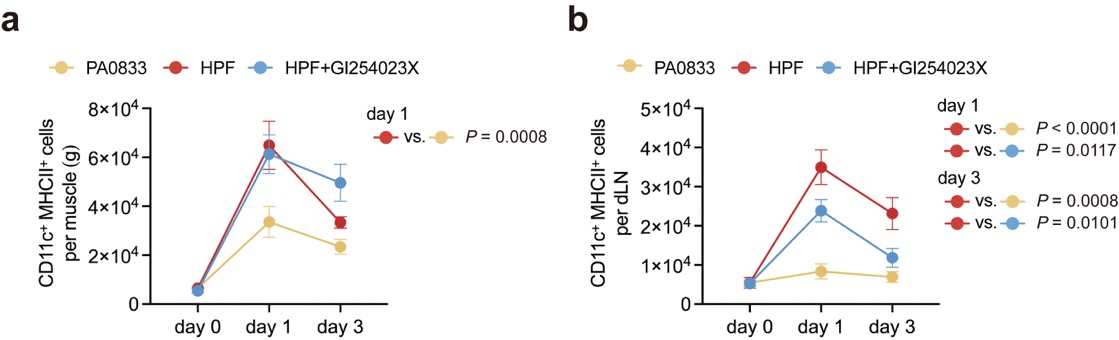
**

**Figure. S2. Hla_H35A_ induces DC accumulation at the injection site and dLN via ADAM10.**

**a** Number of CD11c⁺MHCII⁺ cells per g in muscle at days 0, 1, and 3 postinjection with PA0833, HPF, or HPF with GI254023X (*n* = 5 per group). **b** Number of CD11c⁺MHCII⁺ cells in dLNs at days 0, 1, and 3 postinjection with PA0833, HPF, or HPF with GI254023X (*n* = 5 per group). The data are presented as the means ± s.e.m.s. Statistical significance was tested by one-way ANOVA followed by Tukey’s multiple comparisons test in **a** and **b**. dLN: draining lymph nodes.

**
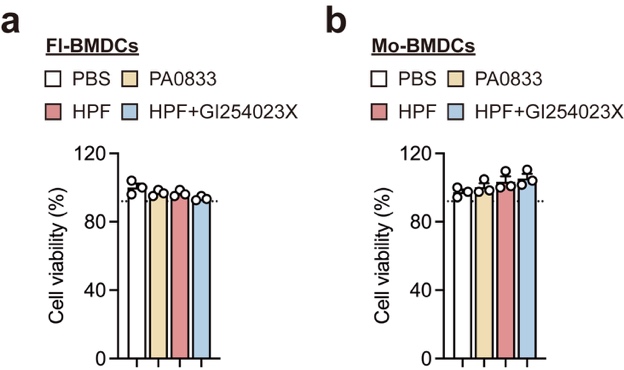
**

**Figure. S3. Different antigenic stimuli do not affect the viability of BMDCs.**

**a** Viability of Fl-BMDCs after 24 h of treatment with PBS, PA0833, HPF, or HPF GI254023X (*n* = 3 per group). **b** Viability of Mo-BMDCs after 24 h of treatment with PBS, PA0833, HPF, or HPF with GI254023X (*n* = 3 per group). Each data point indicates a biological replicate in **a** and **b** Data are presented as the mean ± s.e.m. Statistical significance was tested by one-way ANOVA followed by Tukey’s multiple comparisons test in **a** and **b**. Mo-BMDCs: GM-CSF/IL-4 induced monocyte-like bone marrow dendritic cells.

**
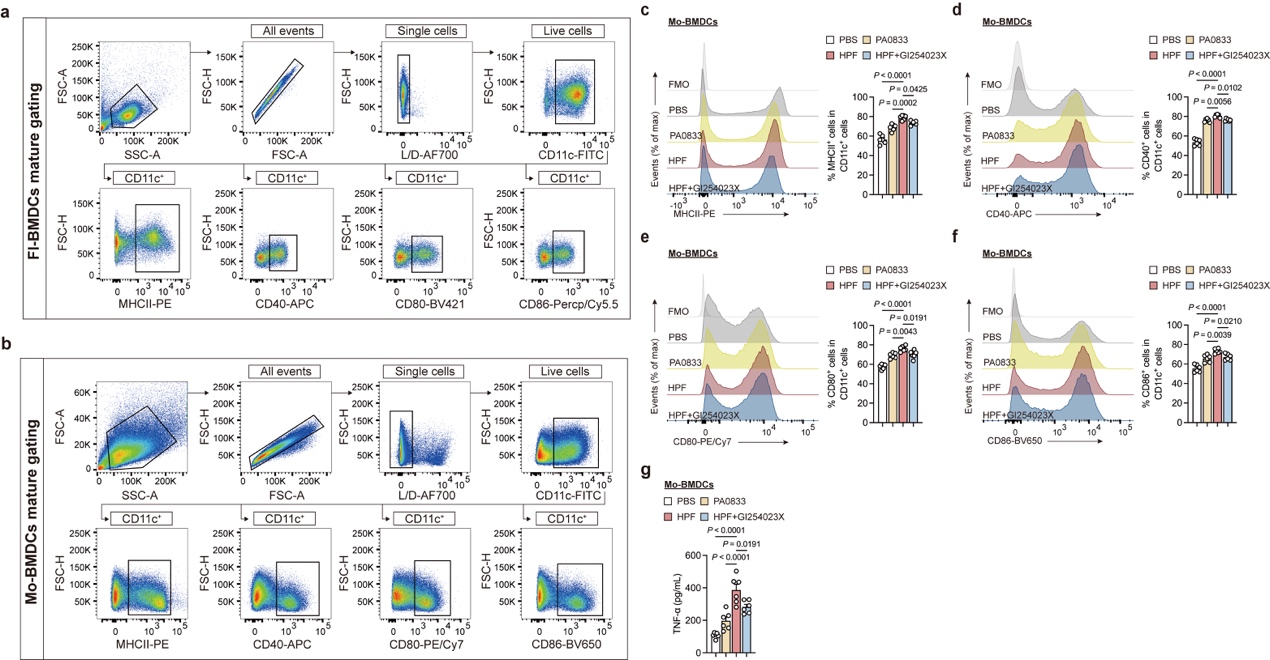
**

**Figure. S4. Hla_H35A_ promotes Mo-BMDC maturation via ADAM10.**

**a** Representative flow plots showing the gating strategies used for Fl-BMDC maturation. **b** Representative flow plots showing the gating strategies used for Mo-BMDC maturation. **c** Representative flow cytometry histogram (left) and quantification (right) of MHCII^+^ cell frequencies gated on CD11c^+^ cells from Mo-BMDCs after 7.5 h of treatment with PBS, PA0833, HPF, or HPF with GI254023X (*n* = 6 per group). The data were pooled from two independent experiments. **d** Representative flow cytometry histogram (left) and quantification (right) of CD40^+^ cell frequencies gated on CD11c^+^ cells from Mo-BMDCs after 7.5 h of treatment with PBS, PA0833, HPF, or HPF with GI254023X (*n* = 6 per group). The data were pooled from two independent experiments. **e** Representative flow cytometry histogram (left) and quantification (right) of CD80^+^ cell frequencies gated on CD11c^+^ cells from Mo-BMDCs after 7.5 h of treatment with PBS, PA0833, HPF, or HPF with GI254023X (*n* = 6 per group). The data were pooled from two independent experiments. **f** Representative flow cytometry histogram (left) and quantification (right) of CD86^+^ cell frequencies gated on CD11c^+^ cells from Mo-BMDCs after 7.5 h of treatment with PBS, PA0833, HPF, or HPF with GI254023X (*n* = 6 per group). The data were pooled from two independent experiments. **g** Serum levels of TNF-α secreted from Mo-BMDCs after 6 h of treatment with PBS, PA0833, HPF, or HPF with GI254023X (*n* = 6 per group). The data were pooled from two independent experiments. Each data point indicates a biological replicate in (**c-g**). The data are presented as the means ± s.e.m.s. Statistical significance was tested via one-way ANOVA followed by Tukey’s multiple comparisons test in (**c-g**).

**
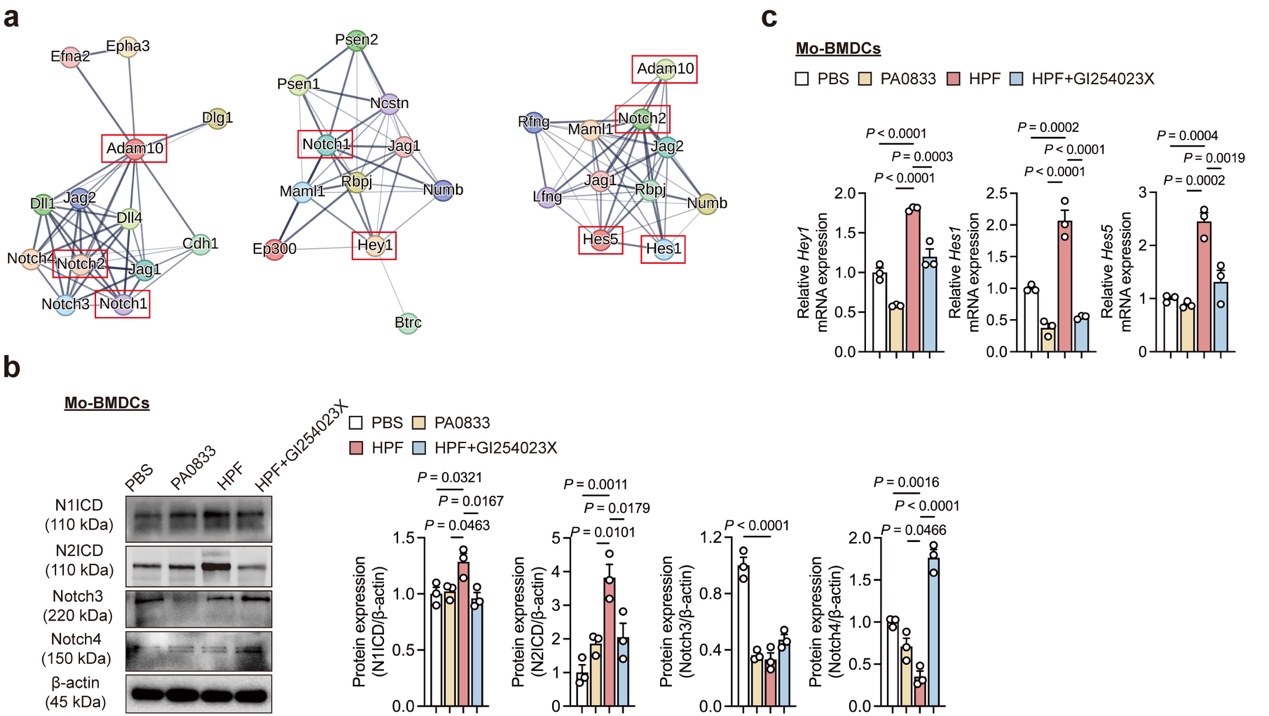
**

**Figure. S5. Hla_H35_ activates Notch signaling via ADAM10 in Mo-BMDCs.**

**a** Protein interaction network of mouse ADAM10, Notch1 and Notch2. **b** Representative western blot (left) and quantification (right) of N1ICD, N2ICD, Notch3, or Notch4 in Mo-BMDCs after 4.5 h of treatment with PBS, PA0833, HPF, or HPF with GI254023X (*n* = 3 per group). **c** Quantification of *Hey1*, *Hes1*, or *Hes5* mRNA expression by qPCR in Mo-BMDCs after 6 h of treatment with PBS, PA0833, HPF, or HPF with GI254023X (*n* = 3 per group). Each data point indicates a biological replicate in **b**. Each data point indicates a biological replicate derived from the average of three technical replicates in **c**. Data are presented as the mean ± s.e.m. Statistical significance was tested via one-way ANOVA followed by Tukey’s multiple comparisons test in **b** and **c**.

**
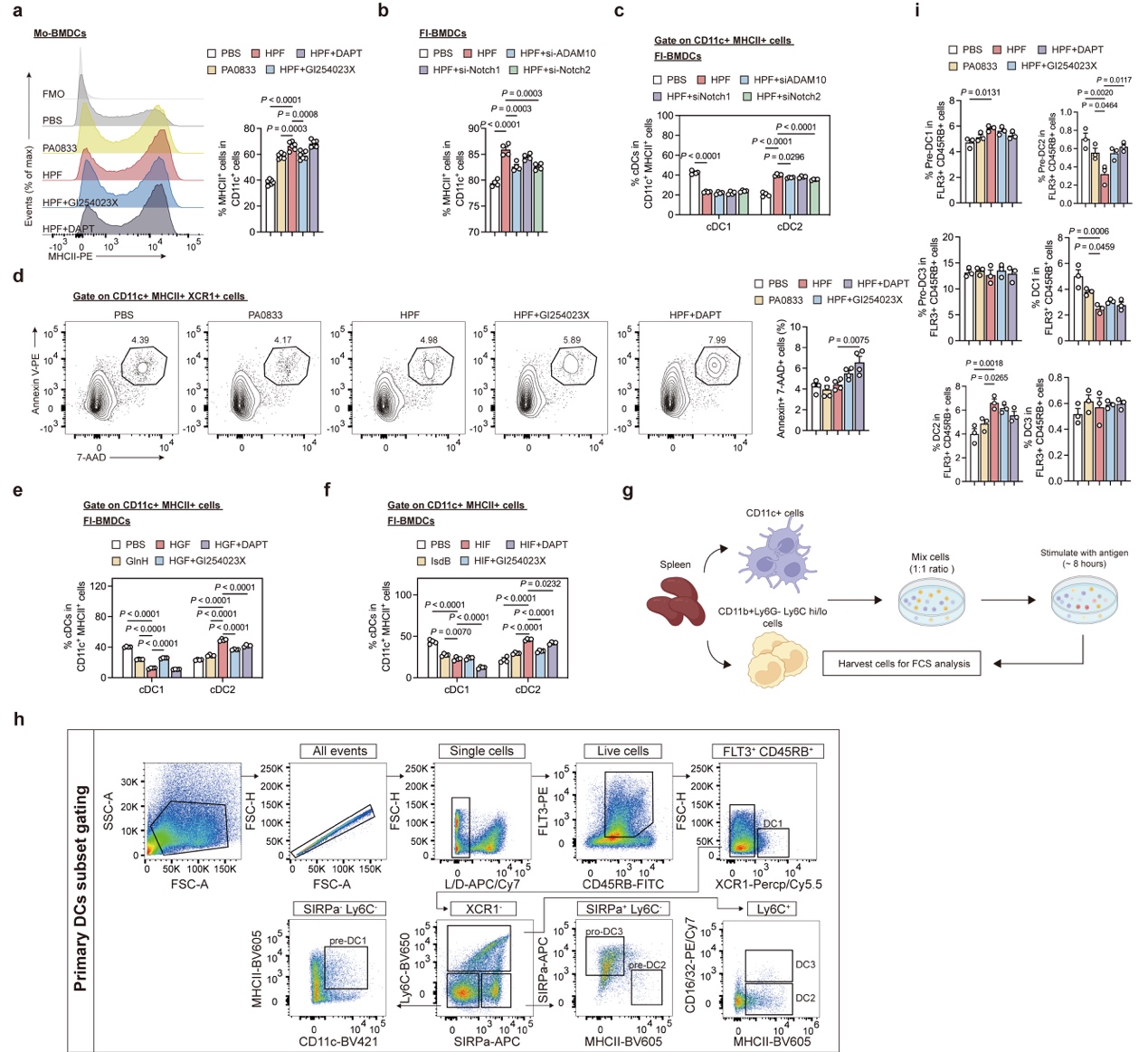
**

**Figure. S6. Hla_H35A_ promotes the differentiation of DCs** **toward the cDC2 subset via ADAM10-Notch signaling.**

**a** Representative flow cytometry histogram (left) and quantification (right) of MHCII^+^ cell frequencies gated on CD11c^+^ cells from Fl-BMDCs after 7.5 h of treatment with PBS, PA0833, HPF, HPF with GI254023X or HPF with DAPT (*n* = 6 per group). The data were pooled from two independent experiments. **b** Representative flow cytometry plots (left) and frequencies (right) of dying (Annexin V^+^ 7-AAD^+^) cDC1s in Fl-BMDCs after 12 h of treatment with PBS, PA0833, HPF, HPF with GI254023X or HPF with DAPT (*n* = 4 per group). **c** Quantification of MHCII^+^ cell frequencies gated on CD11c^+^ cells from Fl-BMDCs after 7.5 h of treatment with PBS, PA0833, HPF, HPF with GI254023X or HPF with DAPT (*n* = 4 per group). **d** Quantification of cDC subset frequencies in Fl-BMDCs after 12 h of treatment with PBS, HPF, HPF with siADAM10, HPF with siNotch1 or HPF with si-Notch2 (*n* = 4 per group). **e** Quantification of cDC subset frequencies in Fl-BMDCs after 12 h of treatment with PBS, GlnH, HGF, HGF with GI254023X or HGF with DAPT (*n* = 4 per group). **f** Quantification of cDC subset frequencies in Fl-BMDCs after 12 h of treatment with PBS, IsdB, HIF, or HIF combined with GI254023X or HIF combined with DAPT (*n* = 4 per group). **g** Experimental design for antigen-induced differentiation of primary dendritic cells from the spleen. **h** Representative flow plots showing the gating strategies used for primary DC differentiation. **i** Quantification of pre-DC1, pre-DC2, pro-DC3, DC1, DC2, and DC3 frequencies in primary DCs after 8 h of treatment with PBS, PA0833, HPF, HPF with GI254023X or HPF with DAPT (*n* = 3 per group). Each data point indicates a biological replicate in (a, c, h, and g). Each data point indicates a biological replicate in (**a-f** and **i)**. The data are presented as the means ± s.e.m.s. Statistical significance was tested via one-way ANOVA followed by Tukey’s multiple comparisons test in (**a-f** and **i)**.

**
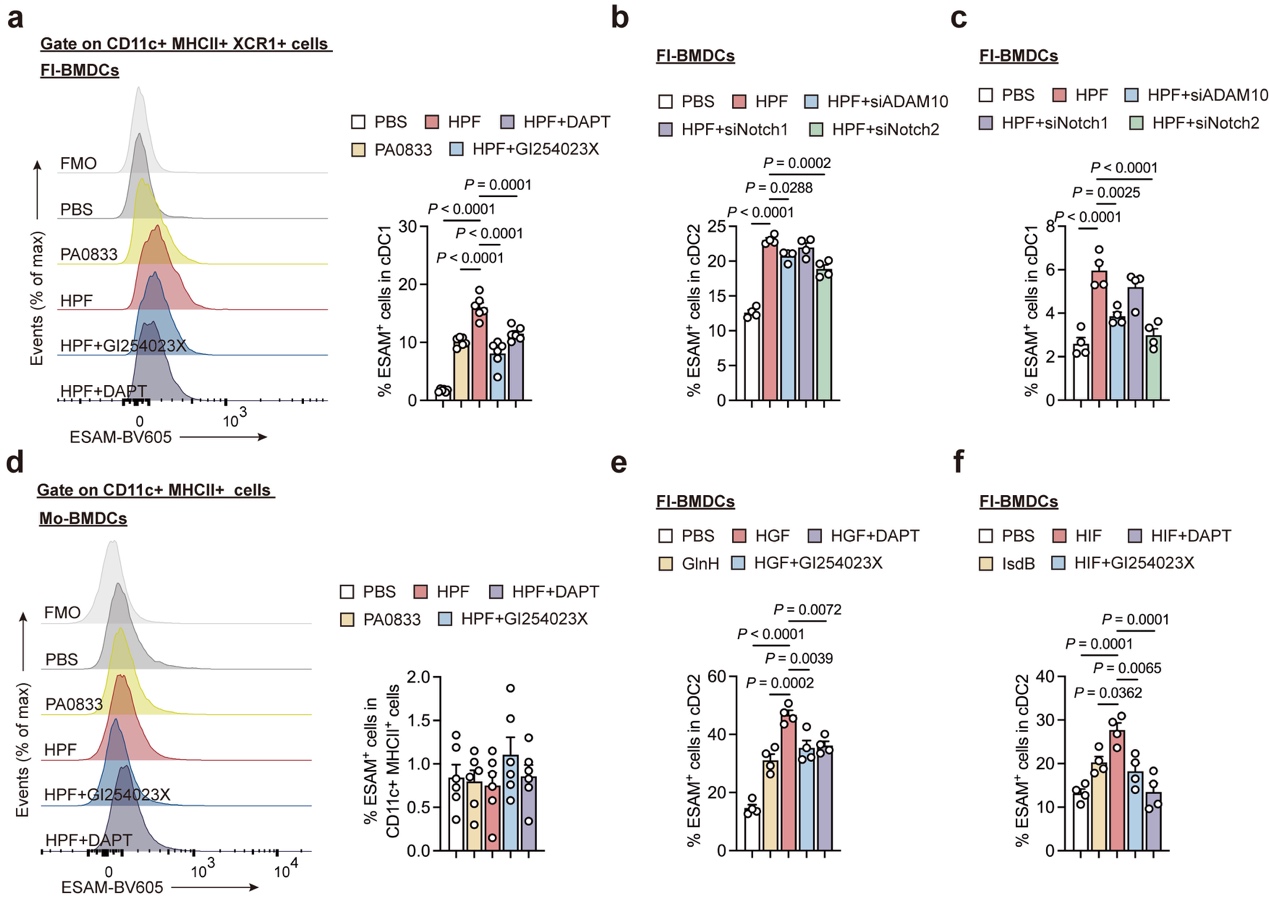
**

**Figure. S7. Hla_H35A_ induces Notch2-dependent cDC2s via ADAM10-Notch2 signaling and is functionally conserved across multiple antigen models.**

**a** Representative flow cytometry histogram (left) and quantification (right) of ESAM^+^ cell frequencies gated on CD11c^+^ MHCII^+^ XCR1^+^ cells in Fl-BMDCs after 12 h of treatment with PBS, PA0833, HPF, HPF with GI254023X or HPF with DAPT (*n* = 6 per group). The data were pooled from two independent experiments. **b** Quantification of ESAM^+^ cell frequencies gated on CD11c^+^ MHCII^+^ SIRPa^+^ cells in Fl-BMDCs after 12 h of treatment with PBS, HPF, HPF with siADAM10, HPF with siNotch1, or HPF with siNotch2 (*n* = 4 per group). **c** Quantification of ESAM^+^ cell frequencies gated on CD11c^+^ MHCII^+^ XCR1^+^ cells in Fl-BMDCs after 12 h of treatment with PBS, HPF, HPF with siADAM10, HPF with siNotch1, or HPF with siNotch2 (*n* = 4 per group). **d** Representative flow cytometry histogram (left) and quantification (right) of ESAM^+^ cell frequencies gated on CD11c^+^ MHCII^+^ cells in Mo-BMDCs after 12 h of treatment with PBS, PA0833, HPF, HPF with GI254023X or HPF with DAPT (*n* = 4 per group). **e** Quantification of ESAM^+^ cell frequencies gated on CD11c^+^ MHCII^+^ SIRPa^+^ cells in Fl-BMDCs after 12 h of treatment with PBS, GlnH, HGF, HGF with GI254023X or HGF with DAPT (*n* = 4 per group). **f** Quantification of ESAM^+^ cell frequencies gated on CD11c^+^ MHCII^+^ SIRPa^+^ cells in Fl-BMDCs after 12 h of treatment with PBS, Isdb, HIF, HIF with GI254023X or HIF with DAPT (*n* = 4 per group). Each data point indicates a biological replicate in (**a-f**). The data are presented as the means ± s.e.m.s. Statistical significance was tested via one-way ANOVA followed by Tukey’s multiple comparisons test in (**a-f**).

**
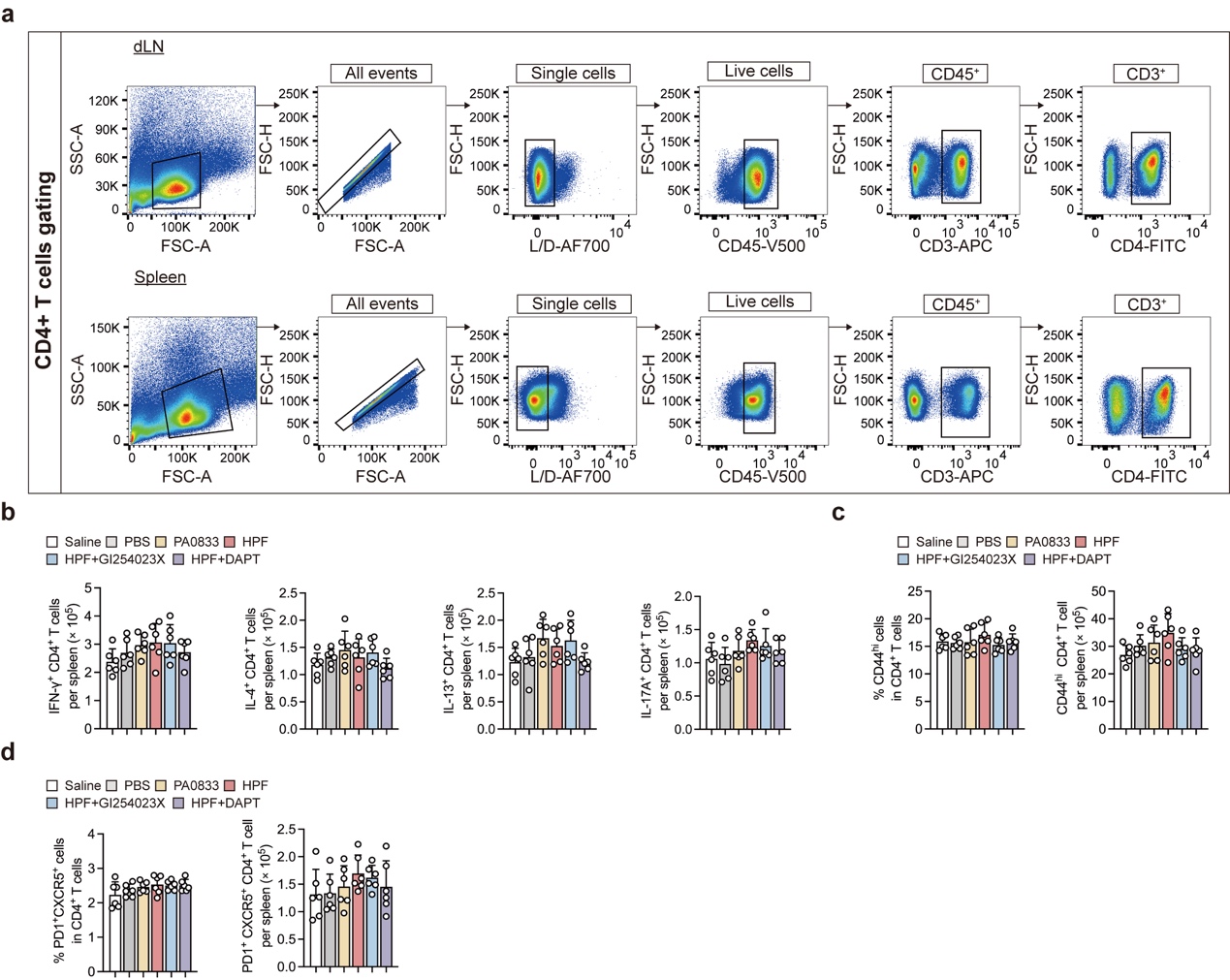
**

**Figure. S8. Hla_H35A_ does not elicit Th17 or Tfh cells in the spleen on day 6 postimmunization via ADAM10-Notch signaling in cDCs.**

**a** CD4^+^ T cells in dLNs and the spleen were gated as CD45^+^CD3^+^CD4^+^ cells. **b** Numbers of cytokine-expressing CD4^+^ T cells in the spleen on day 6 in mice that received Fl-BMDCs after 24 h of treatment with PBS, PA0833, HPF, HPF with GI254023X or HPF with DAPT (*n* = 6 per group). The data were pooled from two independent experiments. **c** Frequencies of CD44^hi^ CD4^+^ T cells (left) and numbers of CD44^+^ CD4^+^ T cells (right) in the spleens of mice that received Fl-BMDCs on day 6 after 24 h of treatment with PBS, PA0833, HPFs, GI254023X HPFs or HPFs plus DAPT (*n* = 6 per group). The data were pooled from two independent experiments. **d** Frequencies of PD1^+^CXCR5^+^ CD44^hi^ CD4^+^ T cells (left) and numbers of PD1^+^CXCR5^+^CD44^hi^ CD4^+^ T cells (right) in the spleen on day 6 in mice that received Fl-BMDCs after 24 h of treatment with PBS, PA0833, HPF, GI254023X or HPF with DAPT (*n* = 6 per group). The data were pooled from two independent experiments. Each data point indicates a biological replicate in (**b**-**d**). The data are presented as the means ± s.e.m.s. Statistical significance was tested via one-way ANOVA followed by Tukey’s multiple comparisons test in (**b**-**d**).


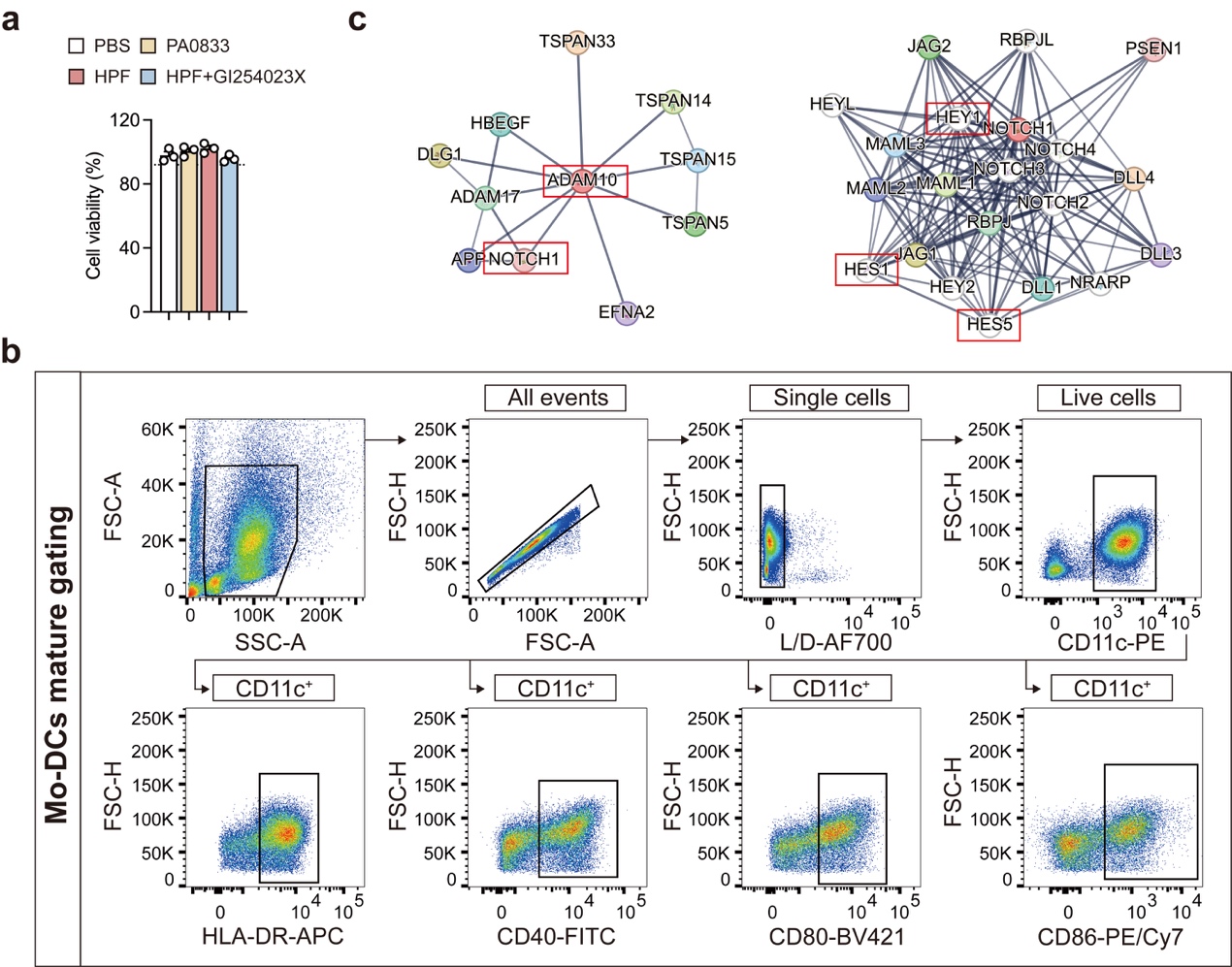


**Figure. S9.** **Hla_H35A_ does not impair cell viability and is involved in ADAM10-Notch signaling in human MoDCs.**

**a** Viability of human MoDCs after 24 h of treatment with PBS, PA0833, HPF, or HPF with GI254023X (*n* = 3 per group). **b** Representative flow plots showing the gating strategies used for human MoDC maturation. **c** Protein interaction network of human ADAM10 and Notch1. The data were pooled from two independent experiments. Each data point indicates a biological replicate in (**a**). The data are presented as the means ± s.e.m.s. Statistical significance was tested via one-way ANOVA followed by Tukey’s multiple comparisons test in (**b**).

**Supplemental Tables**

**Table 1. Sequences for siRNA.**

| **Name** | **Sense** | **Antisense** |
| --- | --- | --- |
| *siNC* | UUCUCCGAACGUGUCACGU | ACGUGACACGUUCGGAGAA |
| *siADAM10* | GCCUCCCAAAGUCUCUCAUAUTT | UUAACAUCAAUCUCACAGCGG |
| *siNotch1* | GCUGUGAGAUUGAUGUUAATT | UUAACAUCUUGCCUGCAGGTG |
| *siNotch2* | GAGCACCUGUGAGCGGAAUAUTT | AUAUUCCGCUCACAGGUGCUCTT |

**Table 2. Antibodies used for flow cytometry.**

| **Antibody** | **Source** | **IDENTIFIER** |
| --- | --- | --- |
| anti-mouse CD11c (FITC) | BD Biosciences | Cat#561045; AB_396683; Clone HL3 |
| anti-mouse CD11c (BV421) | Biolegend | Cat#117343; AB_2563099; Clone N418 |
| anti-mouse CD40 (PE) | Biolegend | Cat#157506; AB_2860731; Clone FGK45 |
| anti-rat CD40 (APC) | BD Biosciences | Cat#558695; AB_1645224; Clone 3/23 |
| anti-mouse CD80 (FITC) | Biolegend | Cat#104705; AB_2291392; Clone 16-10A1 |
| anti-mouse CD80 (BV421) | BD Biosciences | Cat#566285; AB_2737675; Clone 16-10A1 |
| anti-mouse CD86 (APC) | BD Biosciences | Cat#561964; AB_2075114; Clone GL1 |
| anti-mouse CD86 (PerCP) | Biolegend | Cat#105026; AB_893417; Clone GL-1 |
| anti-mouse CD86 (BV510) | BD Biosciences | Cat#564200; AB_2738665; Clone GL1 |
| anti-rat MHCII (BV421) | BD Biosciences | Cat#562564; AB_2716857; Clone M5/114.15.2 |
| anti-rat MHCII (BV510) | BD Biosciences | Cat#742893; AB_2741133; Clone M5/114.15.2 |
| anti-rat MHCII (PE) | BD Biosciences | Cat#562010; AB_396546; Clone M5/114.15.2 |
| anti-mouse MHCII (BV605) | Biolegend | Cat#107639; AB_2565894; Clone M5/114.15.2 |
| anti-mouse/rat XCR1 (PerCP/Cyanine5.5) | Biolegend | Cat#148207; AB_2564364; Clone ZET |
| anti-mouse/rat XCR1 (BV510) | Biolegend | Cat#148218; AB_2565231; Clone ZET |
| anti-rat CD172a (APC) | BD Biosciences | Cat#560106; AB_1645218; Clone P84 |
| anti-mouse CD172a (PerCP/Cyanine5.5) | Biolegend | Cat#144010; AB_2563548; Clone P84 |
| anti-mouse ESAM (BV605) | BD Biosciences | Cat#752438; AB_2917435; Clone: 1G8 |
| anti-mouse ESAM (PE/Cyanine7) | Biolegend | Cat#136212; AB_2860680; Clone: 1G8 |
| anti-rat CD45 (V500) | BD Biosciences | Cat#561487; AB_10697046; Clone 30-F11 |
| anti-mouse CD45 (FITC) | BD Biosciences | Cat#553079; AB_394609; Clone 30-F11 |
| anti-mouse CD45 (V450) | BD Biosciences | Cat# 560501; AB_1645275; Clone 30-F11 |
| anti-mouse CD3 (APC) | Biolegend | Cat#100236; AB_2561456; Clone 17A2 |
| anti-mouse CD3e (BV510) | BD Biosciences | Cat#563024; AB_2737959; Clone 145-2C11 |
| anti-mouse CD4 (FITC) | Biolegend | Cat#100509; AB_312691; Clone GK1.5 |
| anti-mouse CD4 (RB705) | BD Biosciences | Cat#570257; AB_3685618; Clone GK1.5 |
| anti-rat IFN-γ (PerCP-Cy5.5) | BD Biosciences | Cat#560660; AB_1727533; Clone XMG1.2 |
| anti-mouse IFN-γ (FITC) | BD Biosciences | Cat#554411; AB_395375; Clone XMG1.2 |
| anti-mouse IL-4 (BV421) | Biolegend | Cat#504127; AB_2562594; Clone 11B11 |
| anti- mouse IL-4 (PE-Cy7) | BD Biosciences | Cat#560699; AB_1727548 Clone 11B11 |
| anti-rat IL-4 (BV421) | BD Biosciences | Cat#566288; AB_2737889; Clone 11B11 |
| anti-mouse IL-13 (PE) | Biolegend | Cat#159403; AB_2832569; Clone W17010B - |
| anti-mouse IL-17A (PE/Cyanine7) | Biolegend | Cat#506922; AB_2125010; Clone TC11-18H10.1 |
| anti-mouse IL-17A (Alexa Fluor 647) | BD Biosciences | Cat#560184; AB_1645204; Clone TC11-18H10 |
| anti-mouse CD135 (PE) | eBioscience | Cat#12-1351-82; AB_465859; Clone A2F10 |
| anti-mouse CD45RB (FITC) | Biolegend | Cat#103305; AB_313012; Clone C363-16A |
| anti-mouse CD16/32 (PE/Cyanine7) | Biolegend | Cat#101318; AB_2104156; Clone 93 |
| anti-mouse Ly-6C (BV650) | Biolegend | Cat#128049; AB_2800630; Clone HK1.4 |
| anti-mouse PD1 (BV421) | BD Biosciences | Cat#565942; AB_2737668; Clone J43 |
| anti-mouse CXCR5 (PE-Cy7) | BD Biosciences | Cat#560617; AB_1727521; Clone 2G8 |
| anti-mouse/rat XCR1 (BV510) | Biolegend | Cat#148218; AB_2565231; Clone ZET |
| anti-mouse/human CD44 (PerCP/Cyanine5.5) | Biolegend | Cat#103032; AB_2076204; Clone IM7 |
| anti-mouse CD62L (PE) | Biolegend | Cat#161203; AB_2876576; Clone W18021D |
| anti-human CD80 (BV421) | BD Biosciences | Cat#566263; AB_2738632; Clone L307.4 |
| anti-human CD45 (V500) | BD Biosciences | Cat#560779; AB_1937324; Clone HI30 |
| anti-human CD86 (PE-Cy7) | BD Biosciences | Cat#561128; AB_10563077; Clone 2331 (FUN-1) |
| anti-human CD11c (PE) | Biolegend | Cat#301605; AB_314175; Clone 3.9 |
| anti-human HLA-DR (APC) | Biolegend | Cat#307609; AB_314687; Clone L243 |
| anti-human CD40 (FITC) | Biolegend | Cat#334305; AB_1186056; Clone 5C3 |

**Table 3. Primer sequences for qPCR.**

**MOUSE**

| **Genes** | **Forward** | **Reverse** | **Tm (°C)** |
| --- | --- | --- | --- |
| *Hes1* | 5'-AAAGCCTATCATGGAGAAGAGGCG-3' | 5'-GGAATGCCGGGAGCTATCTTTCTT-3' | 61 |
| *Hes5* | 5'-TGCTCAGTCCCAAGGAGAA-3' | 5'-GGCTTTGCTGTGTTTCAGG-3' | 60 |
| *Hey1* | 5'-AGCGTGAGTGGGATCAGT-3' | 5'-GGAGCTGTAGTCTGGGTGA-3' | 61 |
| *Klf4* | 5'-GCGAGTCTGACATGGCTGT-3' | 5'-GTTCCTCACGCCAACGGTTA-3' | 58 |
| *Irf4* | 5'-TGGAGGGATTATGCCCCTGA-3' | 5'-CCTGTCACCTGGCAACCATT-3' | 58 |
| *Ccr7* | 5'-CATGGACCCAGGTGTGCTT-3' | 5'-CATGAGAGGCAGGAACCAGG-3' | 59 |
| *Adam10* | 5'-GAAGATGGTGTTGCCGACAG-3 | 5'-ATTTCCATACTGACCTCCCAGC-3 | 59 |
| *Notch1* | 5'-CACCAGGGTGGTCAGGAAAA-3 | 5'-GGGCAGCGACAGATGTATGA-3 | 60 |
| *Notch2* | 5'-CCGTGGGGCTGAAAAATCTC-3 | 5'-GGGTCATCTTCCGACAGCAA-3 | 58 |
| *Notch3* | 5'-ACTCCTCCTCAGGGAGATGC-3 | 5'-GTGGGGTGAAGCCATCAGG-3 | 61 |
| *Notch4* | 5'-TGGCTATGTCTGCCAGTGTG-3 | 5'-TACAGGTTCCGTGGTTGTGG-3 | 59 |
| *Gapdh* | 5'-GGCATTGTGGAAGGGCTCAT-3' | 5'-AGATCCACGACGGACACATT-3' | 56 |
| *Tlr1* | 5'-GGGTAAGGTTGTCTTGACGGA-3' | 5'-CCAACACGTGGGCTCTTAGT-3' | 60 |
| *Tlr2* | 5'-GCATCCGAATTGCATCACCG-3' | 5'-CCTCTGAGATTTGACGCTTTGT-3' | 56 |
| *Tlr3* | 5'-CTGCGCATATCACAGGCTGA-3' | 5'-ACAGGTGCGTCAACCTCAAA-3' | 58 |
| *Tlr5* | 5'-AATCCCGCTTGGGAGAACAA-3' | 5'-CAGGGGAACCAGGTATGCAG-3' | 56 |
| *Tlr7* | 5'-TGGCTCCCTTCTCAGGATGA-3' | 5'-TTGCTGCGAAGAGTGCATTT-3' | 57 |
| *Tlr11* | 5'-CCACCCCATGCTCAAAGAATC-3' | 5'-GCCAGTCAAGGTAAGGCTCA-3' | 57 |
| *Tlr12* | 5'-TGGCTAGTTCTGCCTTGGTG-3' | 5'-TGAACAAGGCCTGCAGGTAG-3' | 58 |

**HUMAN**

| **Genes** | **Forward** | **Reverse** | **Tm (°C)** |
| --- | --- | --- | --- |
| *Hes1* | ACGACACCGGATAAACCAAAGA | ATGCCGCGAGCTATCTTTCT | 56 |
| *Hes5* | TGAAGCACAGCAAAGCCTTC | AGGCACCACGAGTAGCCTTC | 58 |
| *Hey1* | GCCAGAAAAAGACGGAGAGGAATAA | TGCTCCATTACCTGCTTCTCA | 56 |
| *Tnfα* | GCCCATGTTGTAGCAAACCC | TGAGGTACAGGCCCTCTGAT | 57 |
| *Il6* | TCCTTCTCCACAAACATGTAACAA | TCACCAGGCAAGTCTCCTCA | 56 |
| *Gapdh* | GAAAGCCTGCCGGTGACTAA | GCCCAATACGACCAAATCAGAG | 57 |
